# Cortical Structural Connectivity Alterations and Potential Pathogenesis in Mid-Stage Sporadic Parkinson’s Disease

**DOI:** 10.3389/fnagi.2021.650371

**Published:** 2021-05-31

**Authors:** Xia Deng, Zheng Liu, Qin Kang, Lin Lu, Yu Zhu, Renshi Xu

**Affiliations:** ^1^Department of Neurology, The First Affiliated Hospital of Nanchang University, Nanchang, China; ^2^Department of Neurology, The First Affiliated Hospital of Gannan Medical University, Ganzhou, China; ^3^Department of Neurology, Jiangxi Provincial People’s Hospital, The Affiliated People’s Hospital of Nanchang University, Nanchang, China; ^4^Department of Neurology, The First Affiliated Hospital of Guangzhou Medical University, Guangzhou, China

**Keywords:** Sporadic Parkinson’s disease, magnetic resonance imaging, cortical structural connectivity, pathogenesis, brain

## Abstract

Many clinical symptoms of sporadic Parkinson’s disease (sPD) cannot be completely explained by a lesion of the simple typical extrapyramidal circuit between the striatum and substantia nigra. Therefore, this study aimed to explore the new potential damaged pathogenesis of other brain regions associated with the multiple and complex clinical symptoms of sPD through magnetic resonance imaging (MRI). A total of 65 patients with mid-stage sPD and 35 healthy controls were recruited in this study. Cortical structural connectivity was assessed by seed-based analysis using the vertex-based morphology of MRI. Seven different clusters in the brain regions of cortical thickness thinning derived from the regression analysis using brain size as covariates between sPD and control were selected as seeds. Results showed that the significant alteration of cortical structural connectivity mainly occurred in the bilateral frontal orbital, opercular, triangular, precentral, rectus, supplementary-motor, temporal pole, angular, Heschl, parietal, supramarginal, postcentral, precuneus, occipital, lingual, cuneus, Rolandic-opercular, cingulum, parahippocampal, calcarine, olfactory, insula, paracentral-lobule, and fusiform regions at the mid-stage of sPD. These findings suggested that the extensive alteration of cortical structural connectivity is one of possible pathogenesis resulting in the multiple and complex clinical symptoms in sPD.

## Introduction

Sporadic Parkinson’s disease (sPD) is a neurodegenerative disorder, it is mainly caused by the progressive loss of dopaminergic neurons in the substantia nigra pars compacta projecting to the striatum. The damage of substantia nigra-striatum loops is believed to lead to the hallmarked motor dysfunctions of sPD including tremors, rigidity, bradykinesia, and postural instability (Jankovic, 2008; Göttlich et al., 2013). Apart from the substantia nigra-striatum loops, the deficit of other brain functions has been found in the different stages of sPD. These functional deficits in these brain regions result in multiple and complex clinical symptoms in sPD, which include behavioral disorders, cognitive dysfunction, and autonomic dysfunction such as orthostatic symptoms, incontinence, constipation, hyposmia, and/or dyssomnia (Göttlich et al., 2013; Khoo et al., 2013). However, the relational pathogenesis of brain area damage associated with these multiple and complex clinical symptoms of sPD still remains unclear.

Several neuroimage studies have assessed cortical morphometry using several different methods of magnetic resonance imaging (MRI) and revealed the existence of localized cortical atrophy in the early stages of sPD (Feldmann et al., 2008; Jubault et al., 2011; Gong et al., 2012; Göttlich et al., 2013; Xia et al., 2013; Chen et al., 2015). The motor functional connectivity of the posterior putamen, inferior parietal cortex, and striatum were examined and assessed in sPD patients, which revealed decreased and/or increased functional connectivity in several brain regions of sPD patients (Wu et al., 2009; Helmich et al., 2010; Hacker et al., 2012). A few studies have analyzed the patterns of cortical structural connectivity in sPD patients, and the partial results showed that several functional brain regions found abnormal alterations of cortical structural connectivity (Wu et al., 2009; Helmich et al., 2010; Hacker et al., 2012). However, the specific and typical alterations of cortical structural connectivity in sPD still needs further exploration. Up to now, these multiple and complex clinical symptoms of sPD cannot be completely explained by traditional pathogenesis such as a lesion of the simple typical extrapyramidal circuit between the striatum and substantia nigra, which need to be comprehensively investigated.

Cortical structural connectivity is currently extensively researched in multiple large-scale efforts worldwide, and leads to different definitions with respect to their connections as well as their elements. Perhaps the most promising avenue for defining the connection elements originates from the notion that the individual brain region maintains distinct connection profiles, such as long-range connectivity. These patterns of connectivity determine the functional properties of cortical regions and allow for their anatomical delineation and mapping (Tittgemeyer et al., 2018). The analysis of cortical structural connectivity can measure both global and local connectivity. Therefore, it can be used to assess global brain connectivity or local connectivity between the chosen brain regions (Castellanos et al., 2014; Irimia et al., 2014). Many neurological and psychiatric diseases can be considered as disconnection syndromes related to abnormal brain connectivity. Researchers expect to find and use altered brain connectivity metrics as disease biomarkers, pathological evidence, or treatment targets. For example, some studies have demonstrated specific changes in global and/or local brain connectivity in patients with Alzheimer’s disease, schizophrenia, and focal brain lesions (Stam et al., 2007; Supekar et al., 2008; Gratton et al., 2012; Göttlich et al., 2013). In our previous MRI study, we also found that mid-stage sPD patients presented with reduced cortical thickness in several local clusters by applying the voxel-based morphometry (VBM) method (Deng et al., 2016). Therefore, we speculated that local reduced cortical thickness was possibly implicated in cortical atrophy and/or local cortical structural connectivity alteration in sPD patients (Apostolova and Thompson, 2007; Zarei et al., 2013; Irimia et al., 2014), which might be closely associated with the multiple and complex clinical symptoms and a potential pathogenesis basis of sPD. This study was to investigate the cortical structural connectivity alteration between the chosen cortex regions and potential pathogenesis of sPD through analyzing the alteration of cortical structural connectivity. It aimed to find new imaging alterations in functional magnetic resonance imaging (fMRI), which could explain the relationship between the alteration of cortical structural connectivity and potential pathogenesis of sPD.

In this study, we chose the brain regions of cortical thickness thinning derived from regression analysis using brain size as covariates. These brain regions were used as the seeds of the cortical connectivity assessment, because they were the most extensively altered brain regions and had the most representative cortical thickness thinning among the comparisons between sPD patients and controls (Deng et al., 2016). In total, the seeds consisted of 47 brain regions (Supplementary Table 1). A total of 67 mid-stage sPD patients and 35 healthy controls were recruited in this study. The alteration of their local cortical structural connectivity was assessed between the chosen brain regions according to 47 seed-based correlation analyses (SCA) regarding structural covariates using the VBM of MRI. Results revealed that a larger scale of local alteration of cortical structural connectivity occurred in mid-stage sPD. Our data suggested that the extensive lesion of local cortical structural connectivity was closely associated with the multiple and complex clinical symptoms of sPD, and was a potential pathogenesis of sPD.

## Materials and Methods

### Participants

Participants were acquired from the First Affiliated Hospital of Nanchang University. A total number of 100 sPD patients were recruited for this study. In all of the sPD participants, sPD was diagnosed by three experienced neurologists, the severity of clinical symptoms was assessed according to the Unified Parkinson’s Disease Rating Scale (UPDRS). Thirty-three patients were excluded due to the disqualification of clinical information or MRI data. Sixty-seven mid-stage sPD patients were included in this study based on a UPDRS Part III score of 34.11 ± 2.0 (part III; clinician-scored motor evaluation). All sPD patients were on medication (L-DOPA, agonists). Forty-five control participants were recruited. Ten control subjects were excluded due to disqualification of the clinical information or MRI data. The remaining 35 control subjects showed no sPD signs or any other neurological deficits according to the neurological examination. In order to precisely assess the classification of sPD course, we administered the assessment of modified Hoehn and Yahr and the UPDRS-III scale in sPD patients. The recruited mid-stage sPD patients had to present with all four major clinical signs including tremors, rigidity, bradykinesia, and postural instability, have a better dopaminergic reflection, and have modified Hoehn and Yahr scale and UPDRS-III scale scores of 2.5–3 and 31–45, respectively. The brains of all subjects in this study had normal MRI images in both T1 and T2-weighted images. The relevant demographic and clinical information is summarized in Supplementary Table 2. All procedures have been approved by the ethical committee of the First Affiliated Hospital of Nanchang University and all subjects gave their written informed consent prior to participation. This study was performed in agreement with the Declaration of Helsinki.

### MRI Data Acquisition

High-resolution structural MRI data were acquired from a 3.0 Tesla Siemens Tim Trio MRI scanner, participants were scanned at the Department of Radiology, the First Affiliated Hospital of Nanchang University using a three-dimensional T1-weighted MPRAGE sequence. The scanning parameters were as follows: TR/TE/TI: 1900/2.26/900 ms, flip angle: 9°, slice thickness: 1 mm, 176 slices, field of view 256 mm × 256 mm, acquisition matrix: 256 × 256, voxel size: 1 mm × 1 mm × 1 mm, 8-channels coil. Structural MRI sequences included T1-weighted 3D fast-spoiled gradient-recalled echo images. Other sequences including T2-weighted and FLAIR images were conducted in order to visualize and exclude focal lesions of the cortex or white matter (Deng et al., 2016).

### Image Processing of Cortical Structural Connectivity Using Voxel-Based Morphometry (VBM)

All image processing was performed as per the previous protocol (Deng et al., 2016) and conducted in the State Key Laboratory of Cognitive Neuroscience and Learning and IDG/McGovern Institute for Brain Research, Beijing Normal University, China (Chen et al., 2015). Briefly, the CIVET pipeline was used to measure the cortical thickness and surface on VBM and corticometry. The native T1-weighted MRI images were first linearly aligned into the stereotaxic space and corrected for non-uniformity artifacts using the N3 algorithm (Tohka et al., 2004). The brain image results were then automatically segmented into the gray matter, the white matter, cerebrospinal fluid (CSF), and background by using a partial volume (PV) classification algorithm, in which a trimmed minimum covariance determinant method was applied for estimating the parameters of the PV effect model. The parameter β, controlling the relative strength of the Markov random field, was set to 0.1 (Kim et al., 2005). Next, the inner and outer gray matter surfaces were automatically extracted for each hemisphere using the constrained Laplacian-based automated segmentation with proximities (CLASP) algorithm (Lerch and Evans, 2005). The individual surfaces were further aligned with a surface template to allow comparisons across subjects at corresponding vertices. The cortical surfaces of both the internal and external cortex including 40,962 vertices were automatically extracted by CLASP algorithm.

The cortical surface was inversely transformed into the native space. Cortical thickness was measured between two surfaces on 40,962 vertices per hemisphere between the linked distance in the native space. Cortical thickness was detected by the link method which measured the Euclidean distance between the linked vertices of internal and external surfaces (Lyttelton et al., 2009). The middle cortical surfaces, defined by the geometric center between the internal and external cortical surfaces, were used to calculate the cortical surfaces in the native space. Both thickness and surface maps were further blurred using a 30 mm surface-based smooth diffusion kernel. Vertex-wise sphere-to-sphere warped non-linear surface registration was conducted into the unbiased iterative surface template. The thickness information on native surfaces was transformed into a template after diffusion smoothing by a 20 mm full-width half maximum to increase the signal to noise ratio and improve the detection ability of population changes using the surface registration (Chung et al., 2003). All cortical image processing was conducted by investigators blinded to the patient demographics, disease, and controls. Seven different clusters of cortical thickness thinning derived from the regression analysis using the brain size as covariates in sPD were selected as seeds. Cortical structural connectivity was investigated between the chosen brain regions according to information from 47 SCA regarding structural covariates using the VBM of MRI. The VBM, a technique based on the delineation of the cortex and normalization, can assess cortical atrophy including cortical volume, thickness, surface, and density (Ashburner and Friston, 2000; Deng et al., 2016). These methods have been validated using both manual measurements (Kabani et al., 2001) and a population simulation (Lyttelton et al., 2009), and have been widely applied. The protocol was similar with that of our previous study (Deng et al., 2016).

### Statistical Analysis

We used one-way analysis of variance (ANOVA) with *post hoc* Bonferroni correction to examine the differences in age, education, sPD duration, mini mental state examination (MMSE), Hasegawa dementia scale revised (HDS-R), the forward digit span task (DF), the backward digit span task (DB), semantic verbal fluency test (SVFT), self-rating depression scale (SDS), Hamilton depression scale-17 (HAMD17), Hamilton depression scale-24 (HAMD 24), clock drawing task (CDT), clinical dementia rating (CDR), and the mean cortical thickness values of regions of interest (ROI) between sPD and control groups. A chi-squared test was used to assess the differences in the gender distribution between groups (Deng et al., 2016).

All cortical analyses were performed by the State Key Laboratory of Cognitive Neuroscience and Learning and IDG/McGovern Institute for Brain Research, Beijing Normal University, China. The cortical statistical analysis was performed at a vertex-wise level using an analysis of covariance (ANCOVA) with brain size and cortical surface as covariates and no covariates for comparisons among groups. For all cortical analyses, in order to correct for multiple vertex-wise comparisons, a random field theory (RFT)-based method was applied at the cluster level (Taylor and Adler, 2003), and the cortical clusters surviving a family-wise error (FWE)-corrected *p* < 0.05 were considered as significant. All statistical procedures were implemented using SurfStat^1^. All names of anatomical regions were cited from Anatomical Automatic Labeling (AAL) of the Montreal Neurological Institute (MNI). The significance difference and correlation maps were created as applicable. The maps were corrected for multiple comparisons with permutation analysis at a threshold of *p* < 0.05. The values of cortical thickness and surface were expressed as mean ± SD. A 565comparison of uncorrected cortical thickness between sPD and control was also performed by Student’s t-test with a significance of *p* ≤ 0.05. The uncorrected *p* ≤ 0.05 was the p value that was not corrected by the FWE, the corrected *p* ≤ 0.05 was the p value that was corrected by the FWE. The cortical structural connectivity was expressed by mm, a comparison of cortical structural connectivity between sPD and control was also performed by Student’s t-test with a significance of *p* ≤ 0.05.

## Results

### Clinical Characteristics of Mid-Stage sPD

A total of 67 patients with mid-stage sPD and 35 healthy controls were included in the final investigation and analysis. The details of demographics and disease-related characteristics in both sPD and healthy controls are shown in Supplementary Table 2. The average modified Hoehn and Yahr scale for mid-stage sPD patients was 2.7 ± 0.3, their total UPDRS score (sum of parts I–IV) was 83.17 ± 6.34 and their UPDRS part III score (motor examination) was 34.11 ± 2.0 (Supplementary Table 2).

### Brain Regions of Abnormal Cortical Structural Connectivity Between sPD Patients and Control Group

**FIGURE 1:**
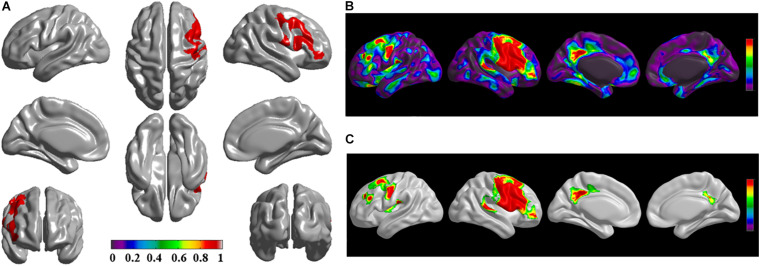
The significantly altered brain regions of cortical structural connectivity in the sPD brain based on seed 1. **(A)** The brain regions of seed 1, the red brain regions represent the cortical thickness thinning regions. **(B)** The significantly altered brain regions in the F-map based on seed 1, the red, and green brain regions represent the brain regions of significant cortical structural connectivity alteration that were not corrected by the family-wise error (FWE). **(C)** The significantly altered brain regions after FWE correction based on seed 1, the red and green brain regions represent the brain regions of significant cortical structural connectivity alteration that were corrected by the FWE.

**FIGURE 2:**
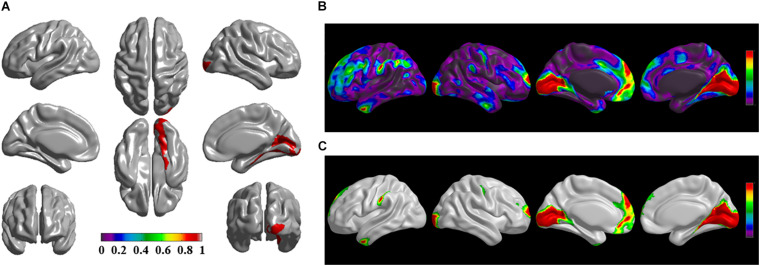
The significantly altered brai n regions of cortical structural connectivity in the sPD brain based on seed 2. **(A)** The brain regions of seed 2, the red brain regions represent the cortical thickness thinning regions. **(B)** The significantly altered brain regions in the F-map based on seed 2, the red, and green brain regions represent the brain regions of significant cortical structural connectivity alteration that were not corrected by the FWE. **(C)** The significantly altered brain regions after FWE correction based on seed 2, the red and green brain regions represent the brain regions of significant cortical structural connectivity alteration that were corrected by the FWE.

**FIGURE 3:**
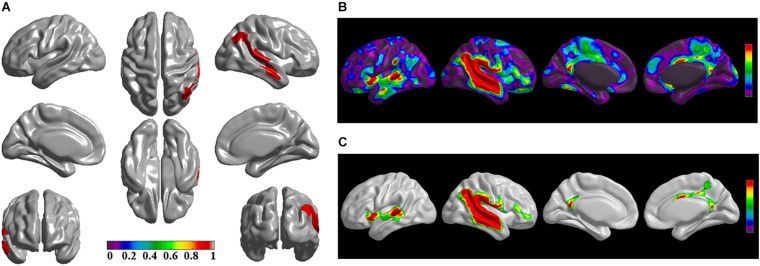
The significantly altered brain regions of cortical structural connectivity in the sPD brain based on seed 3. **(A)** The brain regions of seed 3, the red brain regions represent the cortical thickness thinning regions. **(B)** The significantly altered brain regions in the F-map based on seed 3, the red, and green brain regions represent the brain regions of significant cortical structural connectivity alteration that were not corrected by the FWE. **(C)** The significantly altered brain regions after FWE correction based on seed 3, the red and green brain regions represent the brain regions of significant cortical structural connectivity alteration that were corrected by the FWE.

**FIGURE 4:**
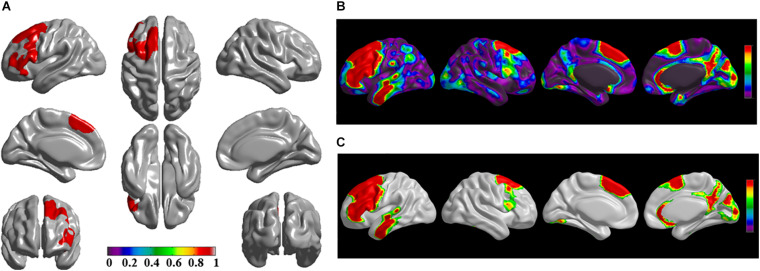
The significantly altered brain regions of cortical structural connectivity in the sPD brain based on seed 4. **(A)** The brain regions of seed 4, the red brain regions represent the cortical thickness thinning regions. **(B)** The significantly altered brain regions in the F-map based on seed 4, the red, and green brain regions represent the brain regions of significant cortical structural connectivity alteration that were not corrected by the FWE. **(C)** The significantly altered brain regions after FWE correction based on seed 4, the red and green brain regions represent the brain regions of significant cortical structural connectivity alteration that were corrected by the FWE.

**FIGURE 5:**
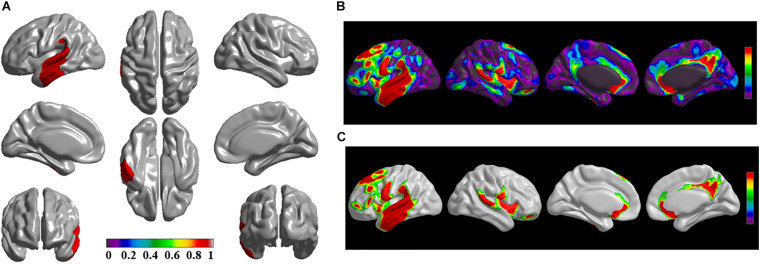
The significantly altered brain regions of cortical structural connectivity in the sPD brain based on seed 5. **(A)** The brain regions of seed 5, the red brain regions represent the cortical thickness thinning regions. **(B)** The significantly altered brain regions in the F-map based on seed 5, the red, and green brain regions represent the brain regions of significant cortical structural connectivity alteration that were not corrected by the FWE. **(C)** The significantly altered brain regions after FWE correction based on seed 5, the red and green brain regions represent the brain regions of significant cortical structural connectivity alteration that were corrected by the FWE.

**FIGURE 6:**
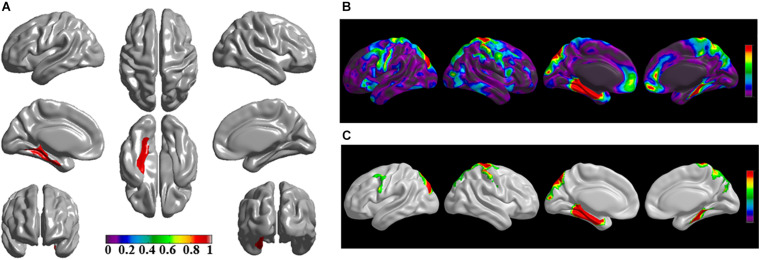
The significantly altered brain regions of cortical structural connectivity in the sPD brain based on seed 6. **(A)** The brain regions of seed 6, the red brain regions represent the cortical thickness thinning regions. **(B)** The significantly altered brain regions in the F-map based on seed 6, the red, and green brain regions represent the brain regions of significant cortical structural connectivity alteration that were not corrected by the FWE. **(C)** The significantly altered brain regions after FWE correction based on seed 6, the red and green brain regions represent the brain regions of significant cortical structural connectivity alteration that were corrected by the FWE.

**FIGURE 7:**
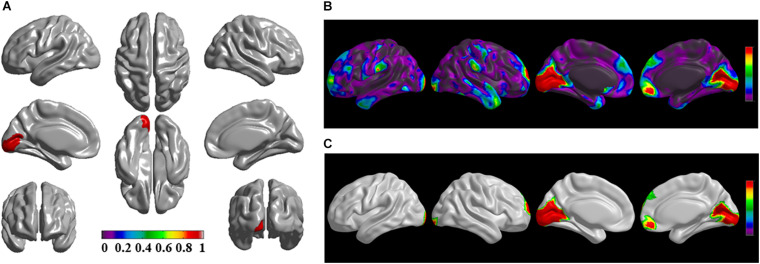
The significantly altered brain regions of cortical structural connectivity in the sPD brain based on seed 7. **(A)** The brain regions of seed 7, the red brain regions represent the cortical thickness thinning regions. **(B)** The significantly altered brain regions in the F-map based on seed 7, the red, and green brain regions represent the brain regions of significant cortical structural connectivity alteration that were not corrected by the FWE. **(C)** The significantly altered brain regions after FWE correction based on seed 7, the red and green brain regions represent the brain regions of significant cortical structural connectivity alteration that were corrected by the FWE.

The brain regions of thinning thickness in the regression analysis using brain size as a covariate were used as the chosen seeds, according to information from 47 SCA regarding structural covariates, cortical structural connectivity between the chosen brain regions was analyzed using the VBM of MRI (Supplementary Table 1 and Figures 1–7A). Results revealed that an extensive alteration in cortical structural connectivity occurred in mid-stage sPD patients, the brain regions of abnormal cortical structural connectivity in the mid-stage sPD patients versus the controls are summarized in Supplementary Tables 3–9. Among them, the brain regions of significantly altered cortical structural connectivity compared with the cortical structural connectivity uncorrected by FWE between sPD and control in seeds 1–7 included the right temporal-pole-sup, the postcentral, the angular, the Rolandic-oper, the cuneus, the cingulum-ant, the occipital-sup, -mid, and –inf, the precuneus and the tectus regions, the left supramarginal, the sup-orb and the temporal-inf regions, the bilateral frontal-sup and -mid, the frontal-sup-medial, the inf-tri, the inf-oper, the inf- and mid-orb, the supp-motor-area, the precentral, the temporal-sup and -mid, the Heschl, the parahippocampal, the parietal-inf, the calcarine, the lingual, and the fusiform regions (Figures 1–7B). The brain regions of significantly altered cortical structural connectivity compared with the cortical structural connectivity corrected by FWE between sPD and control in seed 1 included the right frontal-sup, the mid-, inf-, and sup-orb, the frontal-sup-medial, supp-motor-area regions; the temporal-sup and Heschl regions; the left sup- and mid-tri regions, the paracentral-lobule and the insula regions; the bilateral inf-tri, the inf-oper, the Rolandic-oper, the precentral, the supramargina, the postcentral, the cingulum-post and -mid, and the precuneus regions (Supplementary Table 3 and Figure 1C). Alterations in seed 2 included the right sup- and mid-medial, the precentral, the inf- and mid-cingulum regions; the left frontal-sup, the mid-orb, the rectus, the cingulum-ant, the temporal-pole-mid, the inf- and mid-parietal, and the supramarginal regions; the bilateral frontal-sup-medial, the parahippocampal, the calcarine, the lingual, the fusiform, the precuneus, the cuneus, the occipital-sup, and the postcentral regions (Supplementary Table 4 and Figure 2C). Alternation in seed 3 included the right frontal mid-oper, inf- and mid-orb, inf-tri, the temporal-pole-sup, the parietal-inf, the angular, the supramarginal, the occipital-mid, the Rolandic-oper, the postcentral, the precentral, the paracentral-lobule and the cingulum-ant regions; the left insula and the cuneus regions; the bilateral inf-oper, the temporal-sup, the inf and mid, the Heschl, the cingulum-post and -mid, and the precuneus regions (Supplementary Table 5 and Figure 3C). Alterations in seed 4 included the right parietal-sup, the occipital-sup, the cingulum-post, the parietal-mid and -ant, the precuneus, the cuneus, the rectus, and the olfactory regions; the left temporal-sup and -mid, the Rolandic-oper, the postcentral, and the occipital-inf regions; the bilateral frontal-sup and -mid, the frontal-sup-medial, the mid-, sup-, and inf-orb, the inf-tri, the inf-oper, the supp-motor-area, the precentral, the calcarine, the lingual, and the fusiform regions (Supplementary Table 6 and Figure 4C). Alterations in seed 5 included the right temporal-sup, the cingulum-mid, the cingulum-post, and the precuneus regions; the left frontal-sup and -mid, the frontal-sup-medial, the temporal-pole-mid, -sup, and -inf, the temporal-mid, the supp-motor-area, and the parietal-inf regions; the bilateral frontal-inf-oper, the sup-, inf-, and mid-orb, the inf-tri, the Heschl, the supramarginal, the cingulum-ant, the rectus, the insula, the olfactory, the Rolandic-oper, the postcentral, and the precentral regions (Supplementary Table 7 and Figure 5C). Alternations in seed 6 included the right postcentral and paracentral-lobule regions; the left frontal-mid, the temporal-pole-sup and -mid, the occipital-mid, and the calcarine regions; the bilateral frontal-sup, the parietal-sup, the occipital-sup, the precuneus, the cuneus, the precentral, the parahippocampal, the lingual, and the fusiform regions (Supplementary Table 8 and Figure 6C). Alterations in seed 7 included the right frontal-sup and -mid, the frontal-sup-medial, the mid-orb, the cingulum-mid and -ant, and the rectus regions; the bilateral occipital-sup, -inf, and -mid, the calcarine, the lingual, the precuneus, and the cuneus regions (Supplementary Table 9 and Figure 7C). In general, our result showed that the alteration of cortical structural connectivity comprehensively occurred in the frontal, limbic, temporal, parietal, and occipital lobes, especially in the frontal and limbic lobes, and to smaller extent in the temporal, parietal, and occipital lobes. The majority of alteration regions were symmetrical in both the right and left brain, while the minimum occurred only in the left brain.

## Discussion

The alteration of cortical structural connectivity might be one sign of cortex impairment. For this reason, we studied the alteration of cortical structural connectivity between the chosen brain regions of sPD and the control groups. Results showed that extensive alteration in cortical structural connectivity in many brain regions occurred in mid-stage sPD (Figures 1–7B,C). The brain regions of significantly altered cortical structural connectivity mainly focused on the following brain regions: the frontal cortex including the bilateral frontal-superior and -middle, the inferior-operculum, the inferior-pars triangularis, the inferior- and superior-orbitofrontal, the frontal-superior-medial, the precentral, the rectus, and the supplementary-motor-area cortex, and the right middle-orbitofrontal cortex regions. The temporal cortex alterations included the bilateral temporal-pole-superior, the temporal-superior, -middle, and -inferior, and the Heschl cortex and the left temporal-pole-middle cortex regions. The parietal cortex alterations included the bilateral parietal-inferior and -superior, the supramarginal, the postcentral and the precuneus cortex, the right postcentral-anterior, and the angular cortex regions. The occipital cortex alterations included the bilateral occipital-superior, -inferior, and -middle, the lingual, the cuneus, and the Rolandic-operculum cortex regions. The limbic cortex alterations included the bilateral cingulum-posterior, -middle, and -anterior, the parahippocampal and the calcarine cortex regions. Other cortex alterations included the bilateral olfactory, the insula, the paracentral-lobule, and the fusiform cortex regions (Figures 1–7B,C).

The frontal-sup (superior frontal gyrus) controls self-awareness and laughter (Fried et al., 1998; Goldberg et al., 2006), alteration of cortical structural connectivity in that area might be closely associated with coordination movement disorder and the mask face syndrome in sPD. The frontal-inf-oper (opercular part of the inferior frontal gyrus), also known as BA 44, comprises Broca’s area together with the left-hemisphere BA 45, and is involved in semantic tasks. BA 44 controls phonological and syntactic processing. A recent neuroimage study showed that BA 44 was involved in selective response suppression in go/no-go tasks, therefore, it was believed to play an important role in the suppression of response tendencies. BA 44 was also demonstrated to be related to hand movements (Rizzolatti et al., 2002; Forstmann et al., 2008). Damage to the opercular part of the inferior frontal gyrus caused by the alteration of cortical structural connectivity might generate semantic, phonological, and syntactic processing dysphasia, and a coordination movement disorder in the hands such as tremors and bradykinesia in sPD.

The frontal-mid (middle frontal gyrus) consists of Brodmann areas 9, 10, and 46. Brodmann area 9 is involved in short term memory (Babiloni et al., 2005), verbal fluency (Abrahams et al., 2003), error detection (Chevrier et al., 2007), auditory verbal attention (Nakai et al., 2005), inductive reasoning (Goel et al., 1997), attributing intention (Fink et al., 1999), sustained attention counting a series of auditory stimuli (Shallice et al., 2008), evaluates recency (Zorrilla et al., 1996), overrides automatic responses (Kübler et al., 2006), infers the intention of others (Goel et al., 1995), and comprehends spatial imagery (Knauff et al., 2002). The middle frontal gyrus on the left hemisphere is found to be at least partially responsible for empathy (Farrow et al., 2001), idioms (Maddock and Buonocore, 1997; Lauro et al., 2008), processing pleasant and unpleasant emotional scenes (Lane et al., 1997), self-criticism, and attention to negative emotions (Longe et al., 2010; Kerestes et al., 2012). The middle frontal gyrus on the right hemisphere is involved in attributing intention (Brunet et al., 2000), the theory of mind (Gallagher et al., 2002), suppressing sadness (Kaur et al., 2005), working memory (Zhang et al., 2003), spatial memory (Slotnick and Moo, 2006), recognition (Ranganath et al., 2003), recall (Düzel et al., 2001), the emotions of others (Bermpohl et al., 2006), planning (Fincham et al., 2002), calculation (Rickard et al., 2000), the semantic and perceptual processing of odors (Royet et al., 1999), and attention to positive emotions (Kerestes et al., 2012)^2^. The alteration of cortical structural connectivity in Brodmann area 9 might result in lesions that affect memory, spatial imagery, auditory, intention, emotion, recognition, planning, calculation, and olfactory processes, leading to the corresponding multiple and complex clinical symptoms in sPD. Brodmann area 10’s function is poorly understood. It may perform a domain general function in scheduling operations in working memory, episodic memory, and multiple-task coordination (Andrews et al., 2011). Brodmann area 46 plays a role in sustaining attention and in working memory (Buckner, 1996). The alteration of cortical structural connectivity in Brodmann areas 10 and 46 might damage memory, coordination, and attention, and generate the decline of working and episodic memory, multiple-task coordination, and sustaining attention in sPD.

The frontal-inf-tri (the pars triangularis of the inferior frontal gyrus), also known as Brodmann area 45, comprises Broca’s area together with BA 44 and is active in semantic tasks, such as semantic decision tasks determining whether a word represents an abstract or a concrete entity and generation tasks generating a verb associated with a noun (Gabrieli et al., 1998; Kringelbach, 2005). Damaged cortical structural connectivity of this brain region might produce several types of dysphasia such as those described above in sPD.

The orb (orbitofrontal cortex, OFC) is among the least understood regions of the human brain, but it has been proposed that the OFC is involved in sensory integration, represents the affective value of reinforcers, decision-making and expectation, and adaptive learning (Talati and Hirsch, 2005). Sensing errors, abnormal emotions, and learning ability decline may result from cortical structural connectivity lesions in this area in sPD. The frontal-sup-medial (superior medial frontal gyrus) plays a role in executive mechanisms (Penfield and Welch, 1951). The precentral (precentral gyrus) is the primary motor cortex and controls the voluntary movement of the body. The rectus (or straight gyrus) may be associated with sexual desire. The supp-motor-area (supplementary motor area) controls postural stability during standing or walking, coordinates the temporal sequences of actions, bimanual coordination, and the initiation of internally generated as opposed to stimulus-driven movement (Roland et al., 1980; Halsband et al., 1994; Shima and Tanji, 1998; Serrien et al., 2002; Radua et al., 2010). The alteration of cortical structural connectivity in these brain regions might result in executive and control disorders of voluntary body movement, sexual dysfunction, abnormality of postural and walking stability, and coordination deficits, respectively, in sPD.

The temporal-sup (superior temporal gyrus) is involved in the perception of emotions from facial stimuli (Bigler et al., 2007), auditory processing, language function, and social cognition processes (Dupont, 2002), and is linked to the ability of processing information of the many changeable characteristics of the face. A lesion affecting cortical structural connectivity in this area might be related with the mask face syndrome, a decrease in auditory recognition, dysphasia, cognition impairment, and prosopagnosia in sPD.

The temporal-pole is important in autobiographical memory including taste and olfaction, face recognition, the visual discrimination of two-dimensional pictures, and the mnemonic functions of matching and learning (Acheson and Hagoort, 2013). Dysfunction caused by damaged cortical structural connectivity in these brain regions would generate disorders of taste and olfactory sense, visual recognition deficit, and learning dysfunction in sPD patients.

The exact function of the temporal-mid (middle temporal gyrus) is unknown, but it has been connected with the recognition of known faces and accessing the meaning of words while reading (Hickok et al., 2003). The temporal-inf (inferior temporal gyrus) is an essential region in recognizing patterns, faces, and objects. If cortical structural connectivity is damaged in these brain regions, it would result in the clinical significance of prosopagnosia, alexia, a deficit of semantic memory, and agnosia in sPD.

The temporal-sup (superior temporal gyrus) is involved in the perception of emotions from facial stimuli, auditory processing, and the function of vocabulary language, and potentially develops a sense of language. This region is an important structure in the pathway between the amygdala and prefrontal cortex and is involved in social cognition processes (Yagishita et al., 2008; Hartwigsen et al., 2010). A lesion in this area affecting cortical structural connectivity would produce the mask face syndrome, auditory decline, vocabulary-impaired dysphasia, and the obstacle of social cognition in sPD patients.

The Heschl gyrus (transverse temporal gyrus) is the first cortical structure to process incoming auditory information. The parietal-inf (inferior parietal lobule) is divided into the supramarginal gyrus and the angular gyrus, and is involved in the perception of emotions from facial stimuli, the interpretation of sensory information, and is concerned with language, mathematical operations, and body image (Bigler et al., 2007). The supramarginal gyrus interprets tactile sensory data and is involved in the perception of space and limb location. It also is involved in identifying the postures and gestures of other people (Lacey et al., 2012). The angular gyrus is the part of the brain associated with complex language functions (i.e., reading, writing, and the interpretation of what is written) (Koenigs et al., 2009). The parietal-sup (superior parietal lobule) contains Brodmann areas 5 and 7, is involved in spatial orientation, and receives a great deal of visual input as well as sensory input from the hands (Kjaer et al., 2002; Kamali et al., 2014a,b). The postcentral gyrus is the location of the primary somatosensory cortex. The precuneus gyrus is involved in self-consciousness (Fletcher et al., 1996; Lou et al., 2004), episodic memories, and visuospatial imagery (Cavanna and Trimble, 2006; Valyear et al., 2006). Lesions affecting cortical structural connectivity in these regions produce characteristic symptoms including an auditory lesion, the mask face syndrome, agraphesthesia, astereognosia, hemihypesthesia, alexia, agnosia, logagraphia, the loss of vibration, proprioception, and fine touch, disorder of episodic memories, visuospatial imagery, and hemineglect in sPD patients.

The occipital (occipital gyrus) is divided into several functional visual areas. Each visual area contains a full map of the visual world. The first functional area is the primary visual cortex. It contains a low-level description of local orientation, spatial-frequency, and color properties within small receptive fields. The primary visual cortex projects to the occipital areas of the ventral stream (visual area V2 and visual area V4), and the occipital areas of the dorsal stream (visual area V3, visual area V5, and the dorsomedial area). The ventral stream is known for processing the“wha” in vision, while the dorsal stream handles the “where/how” (Hadland et al., 2003; Bridge, 2011; Wandell and Winawer, 2011). The cuneus (Brodmann area 17) receives visual information from the contralateral superior retina, representing the inferior visual field. The lingula receives information from the contralateral inferior retina, representing the superior visual field (Wandell and Winawer, 2011). Occipital lesions resulting in the alteration of cortical structural connectivity could cause visual hallucinations, color agnosia, and movement agnosia in sPD patients. The Rolandic-oper (post-central operculum or occipital operculum) is responsible for light touch, pain and visceral sensation, and tactile attention (Wandell and Winawer, 2011). The cingulum (cingulate cortex) is an integral part of the limbic system and is involved in emotion formation and procession (Cingulate binds learning, 1997), learning (Stanislav et al., 2013), and memory (Takahashi et al., 2002; Kozlovskiy et al., 2012). It also plays a role in executive function and respiratory control. The parahippocampal (parahippocampal gyrus) plays an important role in the encoding and retrieval of memory (Spaniol et al., 2009; Simmons et al., 2013). The calcarine is the primary visual cortex. The olfactory (olfactory bulb) is involved in the sense of smell. The insula (insular cortex) is involved in consciousness and plays a role in diverse functions usually linked to emotion or the regulation of the body’s homeostasis (Bramão et al., 2010; Gasquoine, 2014). The paracentral-lobule controls the motor and sensory innervations of the contralateral lower extremities. It is also responsible for the control of defecation and urination. The fusiform is linked to various neurological functions such as the processing of color information (Schwarzlose et al., 2005), face and body recognition (Carreiras et al., 2014), and word recognition (Sled and Pike, 1998). Lesions affecting the alteration of cortical structural connectivity in these brain regions were speculated to be associated with a series of neurological phenomena in sPD patients, such as abnormal synesthesia including abnormal light touch, pain, and visceral sensation, dyslexia, prosopagnosia, various visual disorders, autonomic nervous disorders like the disturbance of respiration, body homeostasis, defecation and urination, smell decline, and to a different extent the impairment of emotion, learning, and memory.

Through reviewing the functions of brain regions of significantly altered cortical structural connectivity in sPD, our results revealed that the functional defect of these regions might result in a series of clinical symptoms in mid-stage sPD. Such as the motor symptoms of bradykinesia, a movement coordination deficit, the mask face syndrome, and gait and postural instability. The non-motor symptoms included various disorders of speech, cognition, recognition, emotion, learning, and memory, and to a different extent impairments of smell, taste, auditory, visual, light touch, and pain sensations. Autonomic nervous dysfunctions include abnormal defecation and urination. There would also be a decline in learning, memory, and attention, dysphasia, alexia, agnosia, logagraphia, sexual dysfunction, and so on in sPD patients. The major functions in the regions of significantly altered cortical structural connectivity are strongly related to the generation of multiple and complex clinical symptoms in mid-stage sPD, especially complex non-motor symptoms. The alteration of extensive cortical structural connectivity may have resulted in multiple and complex clinical symptoms in our patients, which suggests that a series of motor and non-motor symptoms in the sPD patients may be derived from the impairment of different brain regions in mid-stage sPD, such as the alteration of cortical structural connectivity. Our results demonstrated that the above described brain regions of significantly altered cortical structural connectivity might result in the impairment of their functions because of cortical damage which led to the clinical-related symptoms in sPD.

The resting-state functional connectivity alteration of the putamen and internal globus pallidus is associated with speech impairment in sPD patients (Manes et al., 2018). This clinically cognitive impairment was related to aberrant intrinsic activity and connectivity within the posterior cingulate, inferior parietal cortex, parahippocampus, entorhinal cortex, sensorimotor cortex (primary motor, pre/post-central gyrus), basal ganglia (putamen, caudate), and posterior cerebellar lobule VII in an sPD patient (Harrington et al., 2017). Up to date, a few investigations into levodopa-induced dyskinesia (LID) have focused on using fMRI. These studies identified the alterations in the brain network activity related to the onset and severity of LID. It was clear from these studies that the LID was associated with the bi-directional altered neuronal firing patterns between the basal ganglia and the neocortex, which led to the overactivation of frontal cortical areas, particularly in the motor, pre-motor, and prefrontal cortices (Cerasa et al., 2012; Cole et al., 2013; Esposito et al., 2013). The fMRI studies consistently demonstrated a linear relationship between the LID and the functional activity, shape, volume, or thickness of brain gray matter (Takeuchi et al., 2010; Zatorre et al., 2012; Fauvel et al., 2014). Although the pathogenesis of sPD symptoms has been looked into using fMRI in the past, the pathogenesis of the symptoms of sPD have not been completely understood yet and are awaiting further deep study.

In our study, we found that the brain regions of significantly altered cortical structural connectivity in mid-stage sPD extensively involved the frontal, temporal, parietal, occipital, and limbic lobe. The extensive alterations of cortical structural connectivity can explain a lot of the multiple and complex symptoms including a few motor and non-motor symptoms in mid-stage sPD. For example, the coordination movement disorder of the face, hands, and body, the abnormality of postural and walking stability (the frontal lobe), depression (the frontal, temporal, and limbic lobe), anxiety (the frontal, temporal, and limbic lobe), dyssomnia (the limbic lobe), cognitive impairment (the frontal and temporal lobe), memory decline (hippocampus), dysfunction of the autonomic nervous system (the parietal lobe), and visual spatial disorder (the occipital lobe). In general, our study provided fMRI evidence conferring that the pathological alteration of brain impairment contributed to some clinical symptoms of sPD. In addition, our study also provided a potential theory of pathogenesis for the multiple and complex clinical symptoms of sPD.

## Limitations

Our study contained a few limitations, such as the small size of the sample, no direct analysis for disease symptom-related measures, and that only a single center was used. We did not perform direct correlation analyses for the potential relationship between brain alterations and clinical symptoms, and only indirectly speculated about their relationship through analyzing the brain function of cortical structural connectivity alteration.

## Conclusion

In summary, our data suggested that the extensive alteration of cortical structural connectivity in mid-stage sPD patients resulted in the dysfunction of corresponding brain regions, generating a series of multiple and complex clinical symptoms. In addition, this study also provided neuroimage information for observing the distributed features of abnormal cortical alteration, understanding the relationship between the brain morphological abnormalities and the clinical symptoms, and reported some novel pathological lesions of the brain and the potential pathogenesis in mid-stage sPD patients. Furthermore, our study identified that the alteration of local cortical structural connectivity is one factor of cortex impairment in mid-stage sPD. Hence, the alteration of local cortical structural connectivity might be used as a diagnostic biomarker for sPD clinical symptoms.

## Data Availability Statement

The original contributions presented in the study are included in the article/Supplementary Material, further inquiries can be directed to the corresponding author.

## Ethics Statement

The studies involving human participants were reviewed and approved by the ethical committee of the First Affiliated Hospital of Nanchang University and all participants gave their written informed consent prior to participate in the study. The study was performed in agreement with the Declaration of Helsinki.

## Author Contributions

RX and XD conceived and designed the experiments, contributed reagents, materials, analysis tools, wrote the manuscript, and processed the figures. XD, ZL, QK, LL, and YZ performed the experiments and analyzed the data. All authors were involved in the drafting, critical revision, final approval of the manuscript for publication, and agreed to be accountable for all aspects of the work in ensuring that questions related to the accuracy or integrity of any part of the work are appropriately investigated and resolved.

## Conflict of Interest

The authors declare that the research was conducted in the absence of any commercial or financial relationships that could be construed as a potential conflict of interest.

## Footnotes

1 http://www.math.mcgill.ca/keith/surfstat/

2 https://en.wikipedia.org/wiki/Brodmann_area_9

## Abbreviations

All abbreviations of names and coordinates of anatomical regions were cited from Anatomical Automatic Labeling of the Montreal Neurological Institute.
