# Supplementary material for: Cortical Structural Connectivity Alterations and Potential Pathogenesis in Mid-Stage Sporadic Parkinson’s Disease
Source: Front Aging Neurosci. 2021 May 31;13:650371. doi: 10.3389/fnagi.2021.650371 (PMC8200851; doi:10.3389/fnagi.2021.650371)
Supplement: Supplementary file 1 [file Table_1.DOCX]

Supplementary Table 1 Brain regions of selected seed

| Brain regions of cortical thickness thinning | Coordinates | | | Mean thickness | | Voxel | Peak F score | P-value |
| --- | --- | --- | --- | --- | --- | --- | --- | --- |
|  | X | Y | Z | HC | sPD |  |  |  |
| **Cluster 1 (Seed 1)** |  |  |  |  |  |  |  |  |
| Frontal-Sup-R | 29.4901 | -10.5107 | 58.981 | 2.912±1.3613 | 2.8188±3.984 | 10 | 9.0341 | 0.088605 |
| Frontal-Inf-Oper-R | 45.0591 | 13.2315 | 23.6988 | 3.2341±1.4757 | 3.138±2.2269 | 270 | 11.7553 | 0.201087 |
| Frontal-Mid-R | 42.1644 | 31.2292 | 18.8666 | 3.0873±1.4474 | 2.9656±3.048 | 557 | 12.7874 | 0.388212 |
| Frontal-Inf-Orb-R | 44.5502 | 43.562 | -2.72275 | 3.2854±1.5231 | 3.1707±2.405 | 41 | 10.885 | 0.250419 |
| Frontal-Mid-Orb-R | 42.7927 | 46..9668 | -1.81597 | 3.2234±1.2054 | 3.1203±2.124 | 60 | 11.3122 | 0.374341 |
| Frontal-Inf-Tri-R | 43.0222 | 30.2529 | 17.4664 | 3.0905±1.1183 | 2.9787±2.331 | 330 | 14.1156 | 0.399803 |
| Rolandic-Oper-R | 59.5857 | -6.24091 | 12.3292 | 3.1205±1.8114 | 3.0124±3.035 | 47 | 9.8802 | 0.318654 |
| Postcentral-R | 42.7618 | -17.2416 | 47.954 | 2.4351±0.9592 | 2.3367±2.368 | 353 | 18.0982 | 0.210631 |
| Precentral-R | 39.9619 | -17.0107 | 47.0979 | 2.706±1.121 | 2.6008±3.498 | 420 | 16.1202 | 0.060772 |
| **Cluster 2 (Seed 2)** |  |  |  |  |  |  |  |  |
| ParaHippocampal-R | 23.4286 | -26.9602 | -20.2916 | 3.2363±1.9298 | 3.0965±4.030 | 185 | 11.0541 | 0.399803 |
| Calcarine-R | 9.62688 | -77.1555 | 11.6999 | 2.6676±1.0073 | 2.5592±2.993 | 629 | 20.3872 | 0.082211 |
| Lingual-R | 21.9604 | -56.5697 | -7.91298 | 2.9457±0.8961 | 2.8425±2.012 | 396 | 11.7836 | 0.313257 |
| Fusiform-R | 24.1216 | -73.1373 | -8.81925 | 2.9642±1.2979 | 2.8739±2.312 | 11 | 8.0218 | 0.398133 |
| Precuneus-R | 19.7339 | -62.5681 | 16.9486 | 3.0264±1.5017 | 2.9401±2.196 | 51 | 13.5812 | 0.172704 |
| Cuneus-R | 19.7435 | -62.2524 | 11.4104 | 2.7444±1.419 | 2.6405±2.913 | 364 | 15.6829 | 0.423608 |
| Occipital-Sup-R | 22.1019 | -100.9825 | -3.11279 | 2.5193±2.009 | 2.3921±3.8834 | 50 | 10.4953 | 0.499524 |
| Occipital-Inf-R | 26.692 | -98.802 | -4.01854 | 2.5924±1.6237 | 2.4828±3.136 | 57 | 9.5471 | 0.499524 |
| Occipital-Mid-R | 25.2149 | -99.6235 | -3.75998 | 2.5933±1.1324 | 2.5011±2.617 | 33 | 9.6531 | 0.275444 |
| **Cluster 3 (Seed 3)** |  |  |  |  |  |  |  |  |
| Parietal-Inf-R | 53.0044 | -50.2854 | 42.8408 | 3.0598±1.8194 | 2.9675±3.270 | 5 | 8.0915 | 0.398133 |
| Angular-R | 46.4373 | -66.9658 | 37.897 | 3.1241±1.2744 | 3.0089±2.097 | 372 | 13.4322 | 0.297979 |
| Heschl-R | 51.8826 | -22.4111 | 8.10232 | 2.7577±1.3399 | 2.6138±3.079 | 133 | 20.8527 | 0.284553 |
| Temporal-Sup-R | 53.4681 | -24.4854 | 9.01416 | 2.9654±1.1474 | 2.813±3.1992 | 938 | 23.3982 | 0.114864 |
| Temporal-Mid-R | 61.9148 | -12.7059 | -16.1903 | 3.4737±1.0343 | 3.3537±2.3797 | 369 | 12.3128 | 0.060772 |
| SupraMarginal-R | 51.1909 | -35.265 | 18.3435 | 2.8329±1.2927 | 2.7254±4.035 | 19 | 10.6551 | 0.060772 |
| Occipital-Mid-R | 43.227 | -61.38322 | 39.2208 | 3.1584±0.7103 | 3.0635±2.524 | 94 | 11.4343 | 0.025177 |
| **Cluster 4 (Seed 4)** |  |  |  |  |  |  |  |  |
| Frontal-Sup-L | -18.405 | 27.4339 | 56.1039 | 3.2203±1.6142 | 3.0701±3.451 | 616 | 14.4461 | 0.365677 |
| Supp-Motor-Area-L | -7.94114 | 22.794 | 59.941 | 3.4617±2.5469 | 3.2852±5.7485 | 408 | 14.4189 | 0.303459 |
| Frontal-Inf-Oper-L | -52.7537 | 13.7529 | 14.5111 | 3.0934±1.1378 | 2.9876±1.992 | 216 | 14.8618 | 0.374341 |
| Frontal-Mid-L | -29.8748 | 7.63778 | 55.0685 | 3.0966±1.3588 | 2.9666±2.216 | 895 | 16.858 | 0.287994 |
| Frontal-Sup-Orb-L | -28.0227 | 54.698 | -0.38587 | 2.9854±1.0589 | 2.8873±2.536 | 1 | 6.9932 | 0.241275 |
| Frontal-Inf-Orb-L | -42.9491 | 28.4152 | -11.7796 | 3.2064±1.5181 | 3.0876±2.974 | 271 | 15.2785 | 0.48645 |
| Frontal-Mid-Orb-L | -42.6875 | 47.1491 | -0.71955 | 3.0357±1.1788 | 2.9413±2.327 | 30 | 7.9455 | 0.473533 |
| Frontal-Sup-Medial-L | -8.21667 | 31.7123 | 55.4035 | 3.565±2.196 | 3.3965±4.366 | 246 | 14.6059 | 0.460781 |
| Frontal-Inf-Tri-L | -52.294 | 17.85 | 19.4534 | 2.9938±1.2021 | 2.8815±2.2496 | 549 | 12.3206 | 0.460967 |
| Precentral-L | -47.9393 | 8.81713 | 19.8343 | 3.1261±1.2709 | 3.028±2.1127 | 20 | 9.6791 | 0.3082 |
| **Cluster 5 (Seed 5)** |  |  |  |  |  |  |  |  |
| ParaHippocampal-L | -28.9172 | -31.9334 | -17.2318 | 3.2524±1.9164 | 3.0802±2.843 | 687 | 18.6054 | 0.18646 |
| Lingual-L | -22.1588 | -58.3478 | -8.18995 | 2.9542±1.0141 | 2.8386±1.648 | 279 | 13.2456 | 0.278245 |
| Fusiform-L | -32.6619 | -30.7991 | -18.836 | 3.2684±1.1662 | 3.1327±2.550 | 238 | 15.5417 | 0.344028 |
| Precuneus-L | -18.5619 | -38.5194 | -0.49079 | 2.8118±2.0257 | 2.7225±2.379 | 18 | 10.1712 | 0.044989 |
| **Cluster 6 (Seed 6)** |  |  |  |  |  |  |  |  |
| Heschl-L | -59.2778 | -12.075 | 2.28861 | 2.7072±1.0744 | 2.5958±3.785 | 91 | 10.086 | 0.027191 |
| Temporal-Sup-L | -57.4669 | -27.1693 | 0.847994 | 2.9608±0.8592 | 2.8093±3.316 | 875 | 19.001 | 0.013619 |
| Temporal-Inf-L | -58.2652 | -24.0822 | -26.477 | 3.3942±1.7706 | 3.1768±2.8402 | 336 | 33.1134 | 0.268733 |
| Temporal-Mid-L | -59.4378 | -19.9477 | -23.2011 | 3.3228±1.0086 | 3.1503±3.238 | 811 | 23.824 | 0.050234 |
| SupraMarginal-L | -59.795 | -31.5237 | 31.9739 | 3.0372±1.5212 | 2.9133±3.400 | 92 | 8.437 | 0.313257 |
| **Cluster 7 (Seed 7)** |  |  |  |  |  |  |  |  |
| Calcarine-L | -7.23996 | -90.2558 | -9.22916 | 2.6311±0.8214 | 2.5039±2.676 | 708 | 20.8572 | 0.046537 |
| Lingual-L | -9.67694 | -84.8585 | -11.3099 | 2.8113±0.6943 | 2.703±2.7056 | 52 | 14.3806 | 0.012616 |
| Cuneus-L | -9.64496 | -70.097 | 13.9467 | 2.44±0.6633 | 2.3507±2.423 | 50 | 12.5979 | 0.020773 |

X, Y and Z were in MNI coordinates. Cortical thickness is expressed by mm.
