# Supplementary material for: Cortical Structural Connectivity Alterations and Potential Pathogenesis in Mid-Stage Sporadic Parkinson’s Disease
Source: Front Aging Neurosci. 2021 May 31;13:650371. doi: 10.3389/fnagi.2021.650371 (PMC8200851; doi:10.3389/fnagi.2021.650371)
Supplement: Supplementary file 2 [file Table_2.DOCX]

Supplementary Table 2. Demographics and disease-related characteristics in sPD and matched controls

| Demographics/Characteristics | sPD  N=67 | Controls  N=35 | Honmo-  geneity of variance test  F-value | t-value | Welch t’-value | P-value |
| --- | --- | --- | --- | --- | --- | --- |
| Demographics  Age, years  Gender, M/F  Education, years  Disease duration, years | 65.31(5.67)  37/30 (M/F)  11.7 (5.50)  8.1(1.17) | 67.3(5.09)  24/11 (M/F)  12.74(5.94)  N/A | 1.24  1.17 | -1.74  χ^2^=1.19  -0.88 |  | 0.08493551  0.2753  0.38096922 |
| Disease severity  Hoehn and Yahr scale  UPDRS Part I- mood and cognition  UPDRS Part II-activities of daily living  UPDRS Part III- motor examination  UPDRS Part IV- medication complications  Total UPDRS score (sum of Parts I-IV) | 2.7(0.3)  8.67(1.37)  28.82(1.59)  34.11(2.0)  11.57(1.38)  83.17(6.34) | N/A  N/A  N/A  N/A  N/A  N/A |  |  |  |  |
| Symptom-dominant side (right/left/double)  Tremor subscoree off (e)  Tremor subscoref on (f)  Webster | 16/44/7  3.37(0.74)  2.26(0.64)  15.67(2.98) | N/A  N/A  N/A  N/A |  |  |  |  |
| MMSE  HDS-R | 15.00(2.47)  16. 80(3.82) | 29.5(0.8)  29.6(2.35) | 9.53  2.64 |  | -43.86  -20.88 | 4.62719E-62  3.07271E-38 |
| DF  DB  SVFT  SDS  HAMD 17  HAMD 24  CDT  CDR  LEDD  PDSI | 5.8(1.51)  3.8(1.44)  14.25(4.40)  66.20(7.16)  34.20(5. 63)  46.6(2.80)  2.25(0.26)  0.49(0.34)  568.97(308.63)  12.2(3.69) | 9.2 (2.1)  6.5(2.1)  17.3(2.8)  28.2(3.4)  2.6(2.4)  3.0(2.6)  3.9(0.3)  0.38(0.33)  N/A  N/A | 1.93  2.13  2.47  4.43  5.50  1.16  1.33  1.33 | 76.49  -28.72 | -8.5  -6.815  -4.26  36.33  39.67  1.98 | 1.55129E-11  9.76169E-09  4.66304E-05  8.48488E-60  1.38241E-62  1.49963E-90  4.18693E-50  0.05160411 |
| Cardinal motor symptoms  Tremor,  Rigidity  Bradykinesia  Postural instability | 67  67  67  67 | N/A  N/A  N/A  N/A |  |  |  |  |

Abbreviation: MMSE=mini mental state examination; HDS-R=Hasegawa dementia scale revised; DF=the forward digit span task; DB=the backward digit span task; SVFT=semantic verbal fluency test; SDS=self-rating depression scale; HAMD=Hamilton depression scale; CDT=clock drawing task; CDR=clinical dementia rating; LEDD=Levodopa (l-dopa) equivalent daily doses; PDSI=the PD screening instrument scores. N/A=not applicable.

Comment: HAMD17 represents the sum of the previous 17 Hamilton anxiety scale item; HAMD24 represents the sum of the previous 24 Hamilton anxiety scale item; Tremor subscoree off (e) represents the sum of the following unified Parkinson’s Disease rating scale (III) item: 20. Tremor subscoref on (f) represents the sum of the following unified Parkinson’s Disease rating scale (III) item: 21. LEDD=[levodopa (×1.2 if catechol-O-methyltransferase (COMT) inhibitor) (×1.2 if 10 mg of selegiline or × 1.1 if 5 mg of selegiline)] + [pramipexole × 400] + [Ropinirole  × 40] + [Cabergoline× 160]+ [pergolide × 200] +  [bromocriptine × 10] +  [lisuride × 160], all doses are in mg.
