# Supplementary material for: Cortical Structural Connectivity Alterations and Potential Pathogenesis in Mid-Stage Sporadic Parkinson’s Disease
Source: Front Aging Neurosci. 2021 May 31;13:650371. doi: 10.3389/fnagi.2021.650371 (PMC8200851; doi:10.3389/fnagi.2021.650371)
Supplement: Supplementary file 3 [file Table_3.DOCX]

Supplementary Table 3 Brain regions of abnormal cortical connectivity in sPD patients versus control in seed 1

| Brain regions of abnormal cortical connectivity | Coordinates | | | Voxel | Peak F  score | Mean cortical  structural connectivity | | P-value |
| --- | --- | --- | --- | --- | --- | --- | --- | --- |
|  | X | Y | Z |  |  | sPD | NC |  |
| **Cluster 1** |  |  |  |  |  |  |  |  |
| Frontal_Sup_R | 29.4405 | -9.66149 | 57.3552 | 513 | 23.9215 | 2.9634±4.0941 | 3.0992±1.5051 | 0.007074* |
| Supp_Motor_Area_R | 15.1682 | 24.0769 | 58.0375 | 89 | 16.1528 | 3.0095±7.5135 | 3.2228±2.5363 | 0.001676* |
| Frontal_Inf_Oper_R | 51.9999 | 11.4201 | 11.6443 | 367 | 87.566 | 3.1777±2.0678 | 3.2669±1.4835 | 0.025354* |
| Frontal_Mid_R | 39.9402 | 10.1319 | 49.1176 | 1355 | 108.6337 | 3.0033±2.408 | 3.0955±1.23794 | 0.486450 |
| Frontal_Sup_Orb_R | 28.2153 | 55.0783 | -1.43741 | 8 | 8.3714 | 2.936±2.1497 | 3.0018±0.7499 | 0.067266 |
| Frontal_Inf_Orb_R | 44.3626 | 43.7541 | -1.64146 | 33 | 13.3997 | 3.1629±2.2901 | 3.277±1.4901 | 0.005961* |
| Frontal_Mid_Orb_R | 38.0295 | 54.8596 | -4.43922 | 165 | 16.4584 | 3.0724±2.0139 | 3.1574±0.8781 | 0.018246* |
| Frontal_Sup_Medial_R | 16.012 | 27.6616 | 56.3079 | 18 | 14.3972 | 3.1628±6.7379 | 3.321±2.1717 | 0.012624* |
| Frontal_Inf_Tri_R | 44.4873 | 23.8326 | 27.3464 | 594 | 68.816 | 3.0414±2.3289 | 3.1409±1.3178 | 0.014046* |
| SupraMarginal_R | 60.0394 | -20.0235 | 32.3603 | 55 | 9.3947 | 2.9006±3.7597 | 2.927±1.1561 | 0.569956 |
| Rolandic_Oper_R | 52.2991 | 8.60619 | 10.9339 | 216 | 78.4802 | 3.1021±2.8452 | 3.1814±2.0578 | 0.088526 |
| Postcentral_R | 57.184 | -4.87377 | 25.8891 | 718 | 73.79 | 2.3743±2.6687 | 2.4483±0.9598 | 0.065825 |
| Precentral_R | 45.9963 | 1.75334 | 41.8643 | 955 | 81.5878 | 2.661±3.5223 | 2.756±1.2076 | 0.039155* |
| Frontal_Sup_L | -23.7256 | 9.35671 | 54.2246 | 285 | 21.8747 | 3.0344±3.7797 | 3.1865±1.8828 | 0.002756* |
| **Cluster 2** |  |  |  |  |  |  |  |  |
| Frontal_Mid_L | -29.7952 | 11.7346 | 52.8508 | 417 | 25.0259 | 2.9916±3.1216 | 3.1301±1.5028 | 0.002592* |
| Postcentral_L | -50.3483 | -10.3251 | 33.6877 | 176 | 17.0674 | 2.1947±2.9492 | 2.2531±0.8433 | 0.152991 |
| Precentral_L | -48.3142 | -10.286 | 37.4583 | 421 | 19.9164 | 2.6045±4.1743 | 2.7087±1.4477 | 0.037354* |
| Cingulum_Post_L | -9.22696 | -48.1665 | 31.0595 | 199 | 17.7132 | 3.4715±2.304 | 3.4828±1.2453 | 0.772417 |
| **Cluster 3** |  |  |  |  |  |  |  |  |
| Cingulum_Mid_L | -7.87583 | -38.1593 | 38.197 | 404 | 18.5152 | 3.2653±2.3581 | 3.293±1.028 | 0.473205 |
| Precuneus_L | -9.72366 | -52.7683 | 36.5277 | 301 | 14.5071 | 3.33±2.0199 | 3.3682±0.9162 | 0.287195 |
| Paracentral_Lobule_L | -9.38547 | -26.2394 | 48.1697 | 54 | 10.1177 | 2.9533±5.0544 | 3.0135±1.7239 | 0.269661 |
| Heschl_R | 55.172 | -19.0163 | 6.45984 | 60 | 13.6124 | 2.5497±3.0549 | 2.706±1.433 | 0.000609* |
| **Cluster 4** |  |  |  |  |  |  |  |  |
| Temporal_Sup_R | 61.1363 | -29.9362 | 10.9752 | 452 | 18.3551 | 2.6461±4.3355 | 2.8248±1.671 | 0.000696* |
| SupraMarginal_R | 52.897 | -36.8547 | 21.579 | 121 | 11.789 | 2.8239±4.2774 | 2.8854±1.3306 | 0.217877 |
| Frontal_Inf_Oper_L | -36.2155 | 2.54604 | 12.3738 | 71 | 14.2991 | 3.5924±3.391 | 3.553±1.0751 | 0.375602 |
| **Cluster 5** |  |  |  |  |  |  |  |  |
| Insula_L | -35.878 | 3.35672 | 12.031 | 199 | 14.78 | 4.0447±5.1793 | 3.9796±1.6709 | 0.236864 |
| SupraMarginal_L | -52.0215 | -23.6601 | 20.0652 | 86 | 20.4279 | 2.9962±2.8228 | 3.0276±1.2955 | 0.461034 |
| Rolandic_Oper_L | -45.8543 | -24.4134 | 19.3617 | 179 | 17.9249 | 3.3439±3.1054 | 3.3385±1.2733 | 0.904724 |
| Postcentral_L | -52.225 | -22.3925 | 19.6792 | 76 | 19.6591 | 3.0534±2.9439 | 3.0818±1.0986 | 0.498076 |
| **Cluster 6** |  |  |  |  |  |  |  |  |
| Cingulum_Post_R | 7.58418 | -48.5777 | 29.4484 | 152 | 14.8493 | 3.5262±2.4685 | 3.5126±0.7484 | 0.719606 |
| Cingulum_Mid_R | 9.29426 | -41.0352 | 36.0581 | 58 | 12.0014 | 3.4179±2.2695 | 3.4154±1.2145 | 0.952275 |
| **Cont**. |  |  |  |  |  |  |  |  |
| Precuneus_R | 5.61694 | -52.882 | 22.0562 | 76 | 12.2261 | 3.5151±2.2059 | 3.5011±0.8895 | 0.704751 |
| **Cluster 7** |  |  |  |  |  |  |  |  |
| Frontal_Sup_L | -29.1034 | 40.3826 | 29.9105 | 54 | 13.7801 | 3.0781±2.7955 | 3.1566±1.3405 | 0.067266 |
| Frontal_Mid_L | -45.5162 | 29.1294 | 28.8976 | 380 | 16.7002 | 2.9996±3.0787 | 3.0984±1.137 | 0.022943* |
| Frontal_Inf_Tri_L | -42.3914 | 28.6158 | 20.0646 | 1 | 7.0983 | 2.8544±2.2937 | 2.9344±1.1243 | 0.041030* |

X, Y and Z were in MNI coordinates. For each cluster, we report the brain regions of the highest peak value. Cortical connectivity is expressed in mm. * indicates a significance of p≤0.05 uncorrected.
