# Supplementary material for: Cortical Structural Connectivity Alterations and Potential Pathogenesis in Mid-Stage Sporadic Parkinson’s Disease
Source: Front Aging Neurosci. 2021 May 31;13:650371. doi: 10.3389/fnagi.2021.650371 (PMC8200851; doi:10.3389/fnagi.2021.650371)
Supplement: Supplementary file 4 [file Table_4.DOCX]

Supplementary Table 4 Brain regions of abnormal cortical connectivity in sPD patients versus control in seed 2

| Brain regions of abnormal cortical connectivity | Coordinates | | | Voxel | Peak F  score | Mean cortical  structural connectivity | | P-value |
| --- | --- | --- | --- | --- | --- | --- | --- | --- |
|  | X | Y | Z |  |  | sPD | NC |  |
| **Cluster 1** |  |  |  |  |  |  |  |  |
| ParaHippocampal_R | 18.7639 | -41.0793 | -9.57631 | 287 | 22.7013 | 2.9836±3.3883 | 3.1033±1.4905 | 0.010734* |
| Calcarine_R | 14.5482 | -68.8233 | 10.8482 | 1080 | 137.9451 | 2.5917±3.1276 | 2.6841±0.9666 | 0.030849* |
| Lingual_R | 10.8363 | -82.4334 | -11.1978 | 898 | 82.6325 | 2.8155±2.2062 | 2.9035±0.8505 | 0.017780* |
| Fusiform_R | 23.2955 | -75.3434 | -9.42291 | 47 | 12.8906 | 2.8945±2.3722 | 2.9722±1.1982 | 0.051604 |
| Precuneus_R | 19.7339 | -62.5681 | 16.9486 | 275 | 30.6862 | 3.0209±2.2355 | 3.0822±1.4073 | 0.126719 |
| Cuneus_R | 12.3672 | -68.3198 | 12.5132 | 733 | 125.232 | 2.6426±2.4167 | 2.712±1.0521 | 0.078113 |
| Occipital_Sup_R | 16.47281 | -101.7559 | -3.945566 | 122 | 41.8435 | 2.4176±3.3372 | 2.5167±1.2953 | 0.029385* |
| Occipital_Inf_R | 25.6305 | -98.9321 | -6.66106 | 140 | 18.5922 | 2.6247±2.8612 | 2.7046±1.6674 | 0.074876 |
| Occipital_Mid_R | 24.2994 | -99.2019 | -7.84047 | 65 | 21.1908 | 2.5061±2.6856 | 2.5864±0.9551 | 0.046058* |
| **Cluster 2** |  |  |  |  |  |  |  |  |
| ParaHippocampal_L | -17.5802 | -39.6956 | -9.17871 | 16 | 8.0795 | 2.8412±2.9989 | 3.0054±2.5081 | 0.001141* |
| Calcarine_L | -8.46087 | -74.4012 | 13.3725 | 1021 | 49.1969 | 2.5082±2.6775 | 2.6137±0.7675 | 0.007486* |
| Lingual_L | -16.8865 | -62.108 | 4.16767 | 546 | 24.5836 | 2.734±2.0091 | 2.8154±0.7961 | 0.021816* |
| Precuneus_L | -14.9075 | -55.8813 | 12.504 | 187 | 13.2044 | 3.0915±2.8036 | 3.1071±1.0775 | 0.704751 |
| Cuneus_L | -7.89157 | -71.5037 | 14.7743 | 409 | 42.6268 | 2.6886±2.7762 | 2.7375±0.8352 | 0.221583 |
| Occipital_Sup_L | -15.82668 | -102.5578 | 1.965368 | 6 | 8.9219 | 2.2932±3.9829 | 2.3097±1.2631 | 0.727075 |
| **Cluster 3** |  |  |  |  |  |  |  |  |
| Frontal_Sup_L | -12.1124 | 52.8448 | 36.7249 | 211 | 12.3581 | 3.11±2.8876 | 3.2284±1.5027 | 0.007919* |
| Rectus_L | -3.81996 | 38.8009 | -20.7187 | 177 | 11.4314 | 2.9463±2.1387 | 2.9884±0.6162 | 0.225334 |
| Frontal_Mid_Orb_L | -7.86154 | 40.146 | -10.5426 | 277 | 14.6912 | 3.2187±2.6537 | 3.2882±1.0106 | 0.084936 |
| Frontal_Sup_Medial_L | -6.05056 | 52.6034 | 33.5187 | 882 | 16.4525 | 3.359±2.5552 | 3.4478±1.5048 | 0.037354* |
| Cingulum_Ant_L | -9.2149 | 42.0534 | -7.15576 | 99 | 13.9071 | 3.4297±3.0946 | 3.4422±1.546 | 0.780056 |
| **Cluster 4** |  |  |  |  |  |  |  |  |
| Frontal_Sup_R | 18.2506 | 64.1185 | 11.7278 | 276 | 19.9761 | 2.9788±2.069 | 3.0178±1.6989 | 0.339371 |
| Frontal_Mid_R | 24.2882 | 56.1395 | 14.4411 | 121 | 11.8522 | 3.012±2.4275 | 3.0331±1.4126 | 0.604213 |
| Frontal_Sup_Medial_R | 12.4178 | 64.4986 | 13.3319 | 209 | 14.9208 | 3.2603±2.189 | 3.3169±1.692 | 0.170664 |
| **Cluster 5** |  |  |  |  |  |  |  |  |
| Postcentral_R | 44.148 | -15.7402 | 41.8075 | 116 | 16.0508 | 2.2169±2.8983 | 2.3136±1.25 | 0.024731* |
| Precentral_R | 41.8424 | -15.9201 | 42.7297 | 118 | 12.5204 | 2.4173±4.1661 | 2.5133±1.4357 | 0.055197 |
| **Cluster 6** |  |  |  |  |  |  |  |  |
| Temporal_Pole_Mid_L | -43.2829 | 9.51227 | -39.1587 | 43 | 12.9557 | 3.7685±3.2683 | 3.8494±1.8087 | 0.088526 |
| Temporal_Inf_L | -47.897 | 0.932741 | -37.3023 | 128 | 12.3475 | 3.6631±1.9001 | 3.74±1.6044 | 0.051604 |
| Temporal_Mid_L | -45.4227 | 5.83164 | -39.0777 | 91 | 15.7469 | 3.5465±2.6293 | 3.6564±1.6176 | 0.011963* |
| Fusiform_L | -30.0177 | 0.289563 | -42.7235 | 3 | 8.2055 | 3.878±4.0117 | 3.8948±2.0416 | 0.742090 |
| **Cluster 7** |  |  |  |  |  |  |  |  |
| **Cont.** |  |  |  |  |  |  |  |  |
| Parietal_Inf_L | -60.1619 | -26.1988 | 36.8622 | 134 | 14.8172 | 2.8505±3.4158 | 2.9586±1.7453 | 0.024731* |
| SupraMarginal_L | -60.5588 | -23.7372 | 34.031 | 96 | 15.6109 | 2.9408±3.4406 | 3.031±1.2372 | 0.048212* |
| Postcentral_L | -59.8486 | -19.17 | 24.5333 | 1 | 6.8987 | 2.979±3.3938 | 3.036±0.9925 | 0.196589 |

X, Y and Z were in MNI coordinates. For each cluster, we report the brain regions of the highest peak value. Cortical connectivity is expressed in mm. * indicates a significance of p≤0.05 uncorrected.
