# Supplementary material for: Cortical Structural Connectivity Alterations and Potential Pathogenesis in Mid-Stage Sporadic Parkinson’s Disease
Source: Front Aging Neurosci. 2021 May 31;13:650371. doi: 10.3389/fnagi.2021.650371 (PMC8200851; doi:10.3389/fnagi.2021.650371)
Supplement: Supplementary file 5 [file Table_5.DOCX]

Supplementary Table 5 Brain regions of abnormal cortical connectivity in sPD patients versus control in seed 3

| Brain regions of abnormal cortical structural connectivity | Coordinates | | | Voxel | Peak F  score | Mean cortical  structural connectivity | | P-value |
| --- | --- | --- | --- | --- | --- | --- | --- | --- |
|  | X | Y | Z |  |  | sPD | NC |  |
| **Cluster 1** |  |  |  |  |  |  |  |  |
| Frontal_Inf_Oper_R | 51.7466 | 9.80734 | 4.6107 | 16 | 7.9235 | 3.1706±2.6123 | 3.2599±1.7447 | 0.042981* |
| Parietal_Inf_R | 53.0044 | -50.2854 | 42.8408 | 3 | 8.1978 | 2.9716±3.2953 | 3.066±1.8133 | 0.048212* |
| Angular_R | 53.4221 | -52.9065 | 35.0674 | 381 | 40.954 | 3.0179±2.2549 | 3.1333±1.3598 | 0.004455* |
| Heschl_R | 53.5429 | -20.701 | 7.29682 | 191 | 154.3425 | 2.6699±3.1577 | 2.803±1.4117 | 0.003619* |
| Temporal_Pole_Sup_R | 51.4844 | 4.12026 | -21.1368 | 8 | 9.0168 | 3.5022±3.6275 | 3.6096±1.7657 | 0.028677* |
| Temporal_Sup_R | 61.1387 | -31.3175 | 4.8957 | 1638 | 186.5961 | 2.9243±2.8233 | 3.0535±1.0167 | 0.002088* |
| Temporal_Inf_R | 64.0719 | -30.6632 | -10.4991 | 132 | 23.9289 | 3.3631±3.9356 | 3.3718±1.333 | 0.857517 |
| Temporal_Mid_R | 51.109 | -42.6674 | 10.2242 | 1355 | 55.2537 | 3.2028±2.1567 | 3.2961±0.9738 | 0.012967* |
| SupraMarginal_R | 52.762 | -35.4889 | 18.4078 | 590 | 98.1092 | 2.9077±3.5129 | 2.955±1.1253 | 0.296249 |
| Occipital_Mid_R | 45.3324 | -70.7103 | 32.9144 | 51 | 10.2717 | 3.0979±2.4743 | 3.1889±0.7703 | 0.017326* |
| Rolandic_Oper_R | 60.6419 | -3.60693 | 12.8707 | 90 | 14.9516 | 3.0267±3.1803 | 3.1243±2.0503 | 0.042981* |
| Postcentral_R | 60.0782 | -11.9908 | 15.2666 | 207 | 18.7076 | 2.9072±3.185 | 2.9911±1.4484 | 0.064409 |
| Precentral_R | 60.8584 | 0.559339 | 17.0594 | 91 | 14.7569 | 2.818±5.615 | 2.9208±2.1984 | 0.081466 |
| **Cluster 2** |  |  |  |  |  |  |  |  |
| Cingulum_Post_R | 9.00942 | -41.3748 | 34.8473 | 8 | 8.222 | 3.4793±2.9919 | 3.4679±1.0509 | 0.787717 |
| Cingulum_Mid_R | 3.91039 | -0.809055 | 30.717 | 415 | 21.6287 | 3.2707±1.7715 | 3.253±0.8225 | 0.597287 |
| Cingulum_Ant_R | 3.898 | 5.84695 | 29.6408 | 36 | 17.3751 | 3.0003±2.595 | 2.926±0.8511 | 0.057707 |
| Precuneus_R | 12.0483 | -42.4573 | 50.6844 | 162 | 10.5132 | 3.0345±3.3876 | 3.0631±1.5089 | 0.536668 |
| Paracentral_Lobule_R | 12.431 | -41.8143 | 54.6345 | 26 | 9.5971 | 2.8287±5.0466 | 2.8441±1.3817 | 0.772417 |
| **Cluster 3** |  |  |  |  |  |  |  |  |
| Heschl_L | -51.8563 | -16.0505 | 5.69451 | 198 | 20.0469 | 2.6361±4.0089 | 2.7349±0.9682 | 0.036481* |
| Temporal_Sup_L | -57.1601 | -33.5658 | 4.30661 | 642 | 27.917 | 2.7404±3.5529 | 2.8965±0.8965 | 0.000589* |
| Temporal_Inf_L | -63.8721 | -33.3783 | -8.22269 | 11 | 9.0092 | 3.1509±4.7973 | 3.2519±2.0373 | 0.067266 |
| Temporal_Mid_L | -53.3994 | -37.9086 | 6.18385 | 374 | 17.8272 | 2.9961±3.1772 | 3.1208±0.8281 | 0.003512* |
| **Cluster 4** |  |  |  |  |  |  |  |  |
| Cingulum_Post_R | 5.1511 | -52.1574 | 18.53811 | 98 | 11.2934 | 3.565±2.8203 | 3.5441±0.8243 | 0.597287 |
| Precuneus_R | 6.48069 | -54.5639 | 15.8625 | 240 | 16.6069 | 3.3984±2.2285 | 3.399±1.1714 | 0.992041 |
| **Cluster 5** |  |  |  |  |  |  |  |  |
| Frontal_Inf_Oper_L | -35.7577 | 4.92394 | 11.8484 | 3 | 7.3354 | 3.7497±4.1337 | 3.6913±1.2667 | 0.229131 |
| Insula_L | -35.7808 | 13.4355 | -8.06356 | 426 | 23.2667 | 4.3351±5.3789 | 4.2987±2.775 | 0.543246 |
| **Cluster 6** |  |  |  |  |  |  |  |  |
| Cingulum_Post_L | -5.21142 | -52.2291 | 20.0397 | 136 | 15.1286 | 3.5287±2.7861 | 3.5274±1.2576 | 0.976127 |
| Cingulum_Mid_L | -8.24926 | -40.0052 | 35.1925 | 3 | 7.1291 | 3.4665±2.6356 | 3.4833±1.5877 | 0.697366 |
| Precuneus_L | -5.30543 | -53.3546 | 19.6358 | 297 | 16.0241 | 3.357±2.2998 | 3.36±0.8555 | 0.936397 |
| Cuneus_L | -15.8779 | -56.3339 | 12.899 | 4 | 8.3054 | 3.1316±3.2728 | 3.1436±0.987 | 0.780056 |
| **Cont.** |  |  |  |  |  |  |  |  |
| **Cluster 7** |  |  |  |  |  |  |  |  |
| Frontal_Mid_R | 42.6875 | 47.1491 | -0.719553 | 24 | 11.168 | 3.0658±2.1016 | 3.1783±1.2752 | 0.004077* |
| Frontal_Inf_Orb_R | 44.1107 | 43.8738 | -0.575719 | 9 | 9.7848 | 3.1214±2.2436 | 3.2438±1.4522 | 0.002929* |
| Frontal_Mid_Orb_R | 41.4609 | 49.8714 | -4.27381 | 76 | 11.7278 | 3.1118±2.1176 | 3.2109±1.1428 | 0.009888* |
| Frontal_Inf_Tri_R | 49.5281 | 33.5035 | 8.68602 | 208 | 12.8192 | 3.0055±2.4406 | 3.1215±1.3131 | 0.005010* |

X, Y and Z were in MNI coordinates. For each cluster, we report the brain regions of the highest peak value. Cortical connectivity is expressed in mm. * indicates a significance of p≤0.05 uncorrected.
