# Supplementary material for: Cortical Structural Connectivity Alterations and Potential Pathogenesis in Mid-Stage Sporadic Parkinson’s Disease
Source: Front Aging Neurosci. 2021 May 31;13:650371. doi: 10.3389/fnagi.2021.650371 (PMC8200851; doi:10.3389/fnagi.2021.650371)
Supplement: Supplementary file 6 [file Table_6.DOCX]

Supplementary Table 6 Brain regions of abnormal cortical connectivity in sPD patients versus control in seed 4

| Brain regions of abnormal cortical connectivity | Coordinates | | | Voxel | Peak F  score | Mean cortical  structural connectivity | | | P-value | |
| --- | --- | --- | --- | --- | --- | --- | --- | --- | --- | --- |
|  | X | Y | Z |  |  | sPD | NC |  | |  |
| **Cluster 1** |  |  |  |  |  |  |  |  | |  |
| Frontal_Sup_L | -21.4708 | 29.347 | 52.4246 | 1043 | 108.7053 | 3.0531±2.6832 | 3.1765±1.392 | 0.004200* | |  |
| Supp_Motor_Area_L | -16.012 | 27.6616 | 56.3079 | 646 | 86.3665 | 3.2599±5.5302 | 3.4181±2.4532 | 0.008374* | |  |
| Frontal_Inf_Oper_L | -51.8112 | 21.8069 | 10.6748 | 277 | 36.0591 | 3.0177±1.8941 | 3.1161±1.1252 | 0.007699* | |  |
| Frontal_Mid_L | -45.027 | 23.9907 | 32.3679 | 1804 | 65.1217 | 2.9854±2.2826 | 3.096±1.2198 | 0.005627* | |  |
| Frontal_Sup_Orb_L | -27.8803 | 54.3887 | 0.689708 | 1 | 7.1982 | 2.8829±2.5255 | 2.9813±1.0743 | 0.014423* | |  |
| Frontal_Inf_Orb_L | -47.2147 | 40.0336 | -5.54156 | 239 | 38.836 | 3.0558±2.9339 | 3.1676±1.588 | 0.014810* | |  |
| Frontal_Mid_Orb_L | -43.2387 | 45.9151 | -1.39846 | 71 | 32.8851 | 3.0184±2.1023 | 3.0962±1.4462 | 0.050452 | |  |
| Frontal_Sup_Medial_L | -16.0796 | 30.1891 | 55.3227 | 407 | 92.0311 | 3.4346±3.6122 | 3.5792±2.0966 | 0.004588* | |  |
| Frontal_Inf_Tri_L | -52.4062 | 29.1422 | 5.10248 | 702 | 84.4739 | 2.9006±2.2075 | 3.0064±1.1844 | 0.007074* | |  |
| Rolandic_Oper_L | -52.7513 | 7.70258 | 13.8577 | 43 | 23.8865 | 3.0757±2.7613 | 3.1321±1.1086 | 0.170664 | |  |
| Postcentral_L | -57.3774 | 5.93817 | 14.0878 | 17 | 18.6561 | 2.9888±4.5708 | 3.0426±1.2418 | 0.287195 | |  |
| Precentral_L | -26.2703 | -2.67436 | 52.9989 | 670 | 40.0142 | 2.8483±3.3942 | 2.9362±1.2571 | 0.053976 | |  |
| **Cluster 2** |  |  |  |  |  |  |  |  | |  |
| Parietal_Sup_R | 12.286 | -63.759 | 60.4424 | 6 | 8.0384 | 2.8713±4.7259 | 2.9313±2.0986 | 0.273973 | |  |
| Cingulum_Post_R | 8.21881 | -48.894 | 29.8413 | 253 | 26.4935 | 3.4862±2.4025 | 3.4759±0.7671 | 0.780056 | |  |
| Cingulum_Mid_R | 7.27529 | -35.9546 | 39.3672 | 171 | 14.0684 | 3.4079±2.046 | 3.3996±1.0315 | 0.818562 | |  |
| Precuneus_R | 6.7695 | -53.2749 | 25.4645 | 925 | 24.1659 | 3.2263±1.6248 | 3.2402±0.9779 | 0.675386 | |  |
| Cuneus_R | 16.7812 | -56.5315 | 11.2703 | 49 | 11.8288 | 2.9997±3.7948 | 3.1016±2.0093 | 0.045012* | |  |
| **Cluster 3** |  |  |  |  |  |  |  |  | |  |
| Frontal_Sup_R | 18.8015 | 12.6777 | 63.8657 | 519 | 42.2609 | 2.9879±4.4125 | 3.1295±1.5696 | 0.006496* | |  |
| Supp_Motor_Area_R | 15.7069 | 13.3645 | 64.3331 | 610 | 38.2753 | 3.2445±5.5023 | 3.3915±2.1378 | 0.012290* | |  |
| Frontal_Inf_Oper_R | 50.726 | 13.3027 | 18.6055 | 226 | 13.4507 | 3.1118±2.3764 | 3.2088±1.5025 | 0.020216* | |  |
| Frontal_Mid_R | 24.5124 | 4.84153 | 53.6165 | 306 | 15.7469 | 2.9816±3.4648 | 3.0953±1.6871 | 0.018246* | |  |
| Frontal_Sup_Medial_R | 16.012 | 27.6616 | 56.3079 | 124 | 25.159 | 3.3175±5.6622 | 3.4297±2.0725 | 0.056440 | |  |
| Cingulum_Mid_R | 8.73351 | 9.16444 | 42.7271 | 2 | 8.5077 | 3.4381±3.4471 | 3.523±1.9063 | 0.081466 | |  |
| Frontal_Inf_Tri_R | 54.0014 | 20.0999 | 17.0854 | 199 | 12.5647 | 2.9775±2.6855 | 3.0766±1.1372 | 0.016881* | |  |
| Precentral_R | 48.7633 | 8.61699 | 18.7557 | 44 | 10.9745 | 3.0752±2.891 | 3.1713±1.6009 | 0.032375* | |  |
| **Cluster 4** |  |  |  |  |  |  |  |  | |  |
| Temporal_Sup_L | -53.7585 | -15.7696 | -7.86064 | 464 | 19.0533 | 2.8212±3.4871 | 2.9926±0.9114 | 0.000162* | |  |
| Temporal_Inf_L | -52.1384 | -4.77142 | -33.3782 | 240 | 25.0446 | 3.2686±2.7244 | 3.4729±1.7593 | <0.0001* | |  |
| Temporal_Mid_L | -53.7585 | -15.7696 | -7.86064 | 464 | 19.0533 | 3.2205±3.4128 | 3.3948±1.1533 | 0.000168* | |  |
| **Cluster 5** |  |  |  |  |  |  |  |  | |  |
| Calcarine_R | 6.69215 | -94.2436 | 5.31328 | 441 | 18.1902 | 2.6635±4.064 | 2.7431±1.1119 | 0.096079 | |  |
| Lingual_R | 9.79592 | -79.1867 | -8.14668 | 85 | 12.0845 | 2.793±3.3332 | 2.8681±0.7656 | 0.078113 | |  |
| Cuneus_R | 5.85283 | -80.8543 | 28.6839 | 415 | 21.9506 | 2.6315±2.9863 | 2.6433±0.8263 | 0.772417 | |  |
| **Cont.** |  |  |  |  |  |  |  |  | |  |
| Occipital_Sup_R | 15.80218 | -102.5097 | 3.458317 | 1 | 6.9839 | 2.384±4.5862 | 2.4684±2.1559 | 0.121919 | |  |
| **Cluster 6** |  |  |  |  |  |  |  |  | |  |
| Rectus_R | 14.6451 | 16.2398 | -18.1263 | 97 | 13.4598 | 3.3485±2.3929 | 3.3087±1.4232 | 0.329451 | |  |
| Frontal_Sup_Orb_R | 16.2255 | 13.7706 | -18.8049 | 55 | 12.991 | 3.5757±2.3186 | 3.539±1.7311 | 0.386384 | |  |
| Frontal_Inf_Orb_R | 18.9723 | 11.7087 | -19.9668 | 11 | 10.2908 | 3.756±2.8592 | 3.7219±2.46 | 0.479357 | |  |
| Frontal_Mid_Orb_R | 4.08463 | 21.2327 | -15.8707 | 76 | 19.2976 | 3.2873±2.2358 | 3.2571±1.7602 | 0.467097 | |  |
| Cingulum_Ant_R | 5.77286 | 39.0317 | 3.16342 | 475 | 26.2714 | 3.3395±2.516 | 3.3262±0.9994 | 0.734570 | |  |
| Olfactory_R | 4.55014 | 15.342 | -15.9403 | 91 | 18.4567 | 3.3637±2.497 | 3.3194±1.5357 | 0.291698 | |  |
| **Cluster 7** |  |  |  |  |  |  |  |  | |  |
| Temporal_Inf_R | 42.977 | -25.1305 | -24.7621 | 15 | 8.5682 | 3.3934±1.8612 | 3.3629±1.3665 | 0.419864 | |  |
| Fusiform_R | 40.3709 | -39.067 | -22.7189 | 225 | 15.9831 | 3.4857±2.0591 | 3.4701±1.1558 | 0.675386 | |  |
| **Cluster 8** |  |  |  |  |  |  |  |  | |  |
| Calcarine_L | -20.0862 | -87.7685 | -13.2293 | 7 | 7.5999 | 2.6454±2.6982 | 2.7147±1.2865 | 0.098047 | |  |
| Lingual_L | -18.2933 | -75.003 | -9.25437 | 235 | 16.5461 | 2.8133±2.2896 | 2.8692±0.9404 | 0.139372 | |  |
| Fusiform_L | -24.3051 | -71.8156 | -8.41825 | 152 | 13.6794 | 3.0247±2.5809 | 3.0552±1.1699 | 0.455016 | |  |
| Occipital_Inf_L | -24.4762 | -88.0244 | -15.1328 | 14 | 8.057 | 2.64±2.8946 | 2.7016±1.3144 | 0.152991 | |  |

X, Y and Z were in MNI coordinates. For each cluster, we report the brain regions of the highest peak value. Cortical connectivity is expressed in mm. * indicates a significance of p≤0.05 uncorrected.
