# Supplementary material for: Cortical Structural Connectivity Alterations and Potential Pathogenesis in Mid-Stage Sporadic Parkinson’s Disease
Source: Front Aging Neurosci. 2021 May 31;13:650371. doi: 10.3389/fnagi.2021.650371 (PMC8200851; doi:10.3389/fnagi.2021.650371)
Supplement: Supplementary file 7 [file Table_7.DOCX]

Supplementary Table 7 Brain regions of abnormal cortical connectivity in sPD patients versus control in seed 5

| Brain regions of abnormal cortical connectivity | Coordinates | | | Voxel | Peak F  score | Mean cortical  structural connectivity | | P-value |
| --- | --- | --- | --- | --- | --- | --- | --- | --- |
|  | X | Y | Z |  |  | sPD | NC |  |
| **Cluster 1** |  |  |  |  |  |  |  |  |
| Parietal_Inf_L | -60.5659 | -31.7536 | 39.8942 | 63 | 12.0778 | 2.9224±3.79 | 3.0419±1.866 | 0.017780* |
| Heschl_L | -56.4861 | -10.099 | 2.46775 | 197 | 44.4236 | 2.6331±4.0222 | 2.7323±0.9643 | 0.035625* |
| Temporal_Pole_Sup_L | -50.7987 | 6.27172 | -24.1809 | 14 | 12.8209 | 3.4233±5.877 | 3.5293±1.2384 | 0.058999 |
| Temporal_Pole_Mid_L | -46.6395 | 9.54902 | -36.5634 | 7 | 9.2421 | 3.6508±4.4656 | 3.7532±1.8422 | 0.052779 |
| Temporal_Sup_L | -55.9389 | -14.734 | -6.31565 | 1315 | 126.5717 | 2.8729±3.3195 | 3.0099±0.7703 | 0.001573* |
| Temporal_Inf_L | -55.2983 | -10.2782 | -29.9686 | 468 | 66.5618 | 3.2085±2.3385 | 3.3894±1.5253 | <0.0001* |
| Temporal_Mid_L | -59.3769 | -8.86294 | -17.0932 | 1460 | 145.4502 | 3.1295±2.9126 | 3.2696±0.8284 | 0.000770* |
| SupraMarginal_L | -60.3246 | -29.294 | 29.7531 | 309 | 38.7915 | 2.9202±2.985 | 3.0238±1.3467 | 0.018722* |
| **Cluster2** |  |  |  |  |  |  |  |  |
| Frontal_Inf_Oper_R | 33.8748 | 16.4262 | 9.86302 | 74 | 21.9753 | 3.6868±2.4185 | 3.7198±1.698 | 0.437233 |
| Rectus_R | 11.2587 | 15.486 | -17.5328 | 121 | 19.925 | 3.3091±2.3524 | 3.2724±1.4136 | 0.365011 |
| Frontal_Sup_Orb_R | 22.5063 | 42.0874 | -17.9587 | 320 | 18.71 | 3.3839±1.1961 | 3.3566±0.9406 | 0.370282 |
| Frontal_Inf_Orb_R | 19.8224 | 11.3164 | -20.1049 | 102 | 16.4313 | 3.7343±2.4806 | 3.692±2.3791 | 0.359787 |
| Frontal_Mid_Orb_R | 25.6922 | 46.2503 | -14.7471 | 66 | 14.6302 | 3.3089±1.582 | 3.339±0.9698 | 0.365011 |
| Frontal_Mid_Orb_R | 4.05476 | 20.0919 | -16.2219 | 62 | 26.0017 | 3.2978±2.3186 | 3.2619±1.7973 | 0.397356 |
| Cingulum_Mid_R | 9.97583 | 29.7722 | 27.7859 | 1 | 7.4103 | 3.522±2.8857 | 3.5723±1.7721 | 0.265397 |
| Insula_R | 33.7548 | 10.6351 | 9.65045 | 792 | 31.9007 | 4.2832±4.0649 | 4.2921±2.7645 | 0.873204 |
| Cingulum_Ant_R | 4.71915 | 31.0134 | -8.87407 | 533 | 29.4225 | 3.3938±2.5003 | 3.396±1.0904 | 0.952275 |
| Frontal_Inf_Tri_R | 32.9793 | 18.4498 | 8.9735 | 37 | 20.6161 | 3.6741±3.2935 | 3.6974±2.1016 | 0.632276 |
| Olfactory_R | 4.31277 | 16.4903 | -15.8065 | 120 | 28.2486 | 3.3773±2.4261 | 3.3459±1.5495 | 0.449043 |
| Rolandic_Oper_R | 36.9055 | -4.33513 | 13.0946 | 27 | 16.7061 | 3.6511±3.4036 | 3.6634±2.0865 | 0.803100 |
| **Cluster 3** |  |  |  |  |  |  |  |  |
| Frontal_Sup_L | -21.443 | 30.9494 | 48.2871 | 698 | 18.932 | 3.0538±3.0595 | 3.1967±1.4783 | 0.001729* |
| Supp_Motor_Area_L | -16.012 | 27.6616 | 56.3079 | 131 | 16.3593 | 3.1018±7.6326 | 3.308±2.5648 | 0.002514* |
| Frontal_Inf_Oper_L | -51.0744 | 8.22771 | 15.257 | 94 | 12.4075 | 2.9833±2.1937 | 3.0878±1.0665 | 0.006496* |
| Frontal_Mid_L | -31.3577 | 20.2854 | 47.1582 | 1123 | 26.5094 | 2.9863±2.4909 | 3.1062±1.2814 | 0.003842* |
| Frontal_Sup_Orb_L | -27.8803 | 54.3887 | 0.689708 | 19 | 10.5591 | 2.8896±2.6008 | 2.982±1.0755 | 0.022943* |
| Frontal_Inf_Orb_L | -43.6006 | 45.1341 | -4.71253 | 14 | 10.7513 | 3.0154±2.5612 | 3.1079±1.3647 | 0.027304* |
| Frontal_Mid_Orb_L | -41.7345 | 49.5162 | -0.447113 | 81 | 15.0657 | 2.9779±2.1391 | 3.0638±1.2882 | 0.028677* |
| Frontal_Sup_Medial_L | -11.6218 | 34.5624 | 54.1866 | 146 | 18.7009 | 3.2878±5.1487 | 3.4745±1.9807 | 0.001141* |
| Frontal_Inf_Tri_L | -52.878 | 27.7433 | 12.4205 | 404 | 17.046 | 2.864±2.2772 | 2.9718±1.156 | 0.006313* |
| Rolandic_Oper_L | -59.823 | -3.40108 | 12.0949 | 74 | 20.2736 | 3.0119±3.1934 | 3.0423±0.9846 | 0.479357 |
| Postcentral_L | -61.1173 | -0.4392 | 16.0846 | 179 | 28.7803 | 2.6412±10.4144 | 2.7403±1.1117 | 0.161612 |
| Precentral_L | -59.4836 | 1.80421 | 22.4175 | 455 | 42.2033 | 2.8032±4.0505 | 2.8962±1.358 | 0.057707 |
| **Cluster 4** |  |  |  |  |  |  |  |  |
| Cingulum_Post_R | 8.2188 | -48.894 | 29.8413 | 214 | 26.8293 | 3.4952±2.2953 | 3.4877±0.7421 | 0.834095 |
| **Cont.** |  |  |  |  |  |  |  |  |
| Cingulum_Mid_R | 8.93205 | -40.2667 | 36.9081 | 410 | 20.7116 | 3.2847±1.829 | 3.2684±0.8365 | 0.632276 |
| Cingulum_Ant_R | 3.91018 | 7.96763 | 28.6983 | 15 | 9.9914 | 2.9334±2.0475 | 2.8559±0.7367 | 0.027983* |
| Precuneus_R | 8.03111 | -55.5592 | 35.8814 | 640 | 25.0579 | 3.3049±1.729 | 3.325±1.0125 | 0.563219 |
| **Cluster 5** |  |  |  |  |  |  |  |  |
| Heschl_R | 51.8826 | -22.4111 | 8.10232 | 82 | 18.9143 | 2.5756±3.0499 | 2.7302±1.3816 | 0.000651* |
| Temporal_Sup_R | 60.8172 | -34.9381 | 14.4775 | 768 | 33.7203 | 2.7394±3.803 | 2.9008±1.364 | 0.000969* |
| SupraMarginal_R | 52.762 | -35.4889 | 18.4078 | 70 | 17.8514 | 2.8061±4.4399 | 2.9008±1.4722 | 0.064409 |
| **Cluster 6** |  |  |  |  |  |  |  |  |
| Rectus_L | -3.79928 | 22.7336 | -18.9546 | 52 | 14.6589 | 3.3196±2.1792 | 3.2617±0.9443 | 0.119574 |
| Frontal_Sup_Orb_L | -16.503 | 12.7579 | -18.3374 | 27 | 10.8761 | 3.5215±3.138 | 3.4419±1.5714 | 0.081466 |
| Frontal_Inf_Orb_L | -19.4628 | 9.99093 | -18.8244 | 2 | 7.9821 | 3.6323±3.9211 | 3.5388±1.9789 | 0.067266 |
| Frontal_Mid_Orb_L | -4.64567 | 25.5878 | -14.0182 | 26 | 17.0017 | 3.2969±2.4408 | 3.2967±1.3462 | 1.000000 |
| Frontal_Sup_Medial_L | -9.75738 | 44.5722 | 4.36852 | 21 | 9.9137 | 3.5238±3.0635 | 3.5574±2.0432 | 0.479357 |
| Cingulum_Ant_L | -5.09828 | 37.0039 | -3.48402 | 376 | 27.288 | 3.3366±2.9623 | 3.2949±1.3215 | 0.334387 |
| Olfactory_L | -4.31277 | 16.4903 | -15.8065 | 152 | 31.8654 | 3.3103±2.3778 | 3.2396±1.0397 | 0.070229 |
| **Cluster 7** |  |  |  |  |  |  |  |  |
| Rolandic_Oper_R | 60.6419 | -3.60693 | 12.8707 | 23 | 15.0291 | 2.9171±3.8071 | 3.0351±2.3084 | 0.024122* |
| Postcentral_R | 62.7194 | -8.34434 | 16.5862 | 139 | 23.3737 | 2.7334±3.6806 | 2.8392±1.4379 | 0.027304* |
| Precentral_R | 60.7542 | -2.01428 | 19.3462 | 113 | 18.5009 | 2.7522±5.4864 | 2.854±2.0695 | 0.078113 |
| **Cluster 8** |  |  |  |  |  |  |  |  |
| Frontal_Inf_Oper_L | -35.0033 | 8.46452 | 11.1256 | 26 | 10.4422 | 3.6811±3.5107 | 3.654±1.263 | 0.556521 |
| Insula_L | -36.9388 | 11.3993 | -6.91522 | 470 | 18.8575 | 4.3057±5.3126 | 4.2661±2.5201 | 0.498076 |

X, Y and Z were in MNI coordinates. For each cluster, we report the brain regions of the highest peak value. Cortical connectivity is expressed in mm. * indicates a significance of p≤0.05 uncorrected.
