# Supplementary material for: Cortical Structural Connectivity Alterations and Potential Pathogenesis in Mid-Stage Sporadic Parkinson’s Disease
Source: Front Aging Neurosci. 2021 May 31;13:650371. doi: 10.3389/fnagi.2021.650371 (PMC8200851; doi:10.3389/fnagi.2021.650371)
Supplement: Supplementary file 8 [file Table_8.DOCX]

Supplementary Table 8 Brain regions of abnormal cortical connectivity in sPD patients versus control in seed 6

| Brain regions of abnormal cortical connectivity | Coordinates | | | Voxel | Peak F  score | Mean cortical  structural connectivity | | P-value |
| --- | --- | --- | --- | --- | --- | --- | --- | --- |
|  | X | Y | Z |  |  | sPD | NC |  |
| **Cluster 1** |  |  |  |  |  |  |  |  |
| Frontal_Sup_R | 22.0405 | -10.8633 | 67.8087 | 25 | 10.4843 | 2.6836±5.7464 | 2.7534±2.3239 | 0.240800 |
| Parietal_Sup_R | 16.3533 | -38.7777 | 74.3686 | 436 | 16.1193 | 2.6895±3.5715 | 2.7355±1.1989 | 0.314933 |
| Precuneus_R | 14.6887 | -37.9937 | 75.5046 | 494 | 20.1199 | 2.8948±3.3252 | 2.9299±1.2019 | 0.431397 |
| Cuneus_R | 10.7643 | -75.2642 | 26.7149 | 186 | 13.3417 | 2.5678±2.9543 | 2.5881±0.8591 | 0.618174 |
| Occipital_Sup_R | 19.0825 | -79.8161 | 44.0841 | 111 | 8.9769 | 2.6268±3.8878 | 2.6659±1.2987 | 0.380844 |
| Postcentral_R | 14.5616 | -33.4525 | 74.3204 | 648 | 27.6141 | 2.1263±3.2815 | 2.1667±1.0078 | 0.354611 |
| Paracentral_Lobule_R | 14.2906 | -34.507 | 74.984 | 223 | 26.8499 | 2.2737±7.7001 | 2.2786±1.4604 | 0.936397 |
| Precentral_R | 16.4653 | -28.4647 | 70.5283 | 200 | 21.5598 | 2.2606±6.1299 | 2.2837±1.544 | 0.690010 |
| **Cluster 2** |  |  |  |  |  |  |  |  |
| ParaHippocampal_L | -28.8723 | -31.006 | -17.965 | 1128 | 335.6431 | 3.209±3.789 | 3.3564±1.8466 | 0.003619* |
| Temporal_Pole_Sup_L | -27.4824 | 1.0619 | -24.4038 | 64 | 16.5415 | 4.5554±7.9043 | 4.6033±3.7776 | 0.504402 |
| Temporal_Pole_Mid_L | -24.9674 | -3.15556 | -34.4265 | 3 | 9.2231 | 4.2288±6.3431 | 4.3055±2.9081 | 0.229131 |
| Lingual_L | -17.3415 | -40.7306 | -8.77133 | 292 | 120.1084 | 2.8235±1.9751 | 2.9321±1.2933 | 0.004865* |
| Fusiform_L | -32.9211 | -27.5615 | -21.1985 | 467 | 224.6816 | 3.2607±2.1068 | 3.3631±0.988 | 0.006313* |
| Precuneus_L | -15.9711 | -40.573 | -3.02622 | 136 | 41.1018 | 2.8275±2.7896 | 2.8777±1.7865 | 0.265397 |
| Cuneus_L | -18.2511 | -48.0347 | 0.078201 | 10 | 9.3227 | 2.8324±3.6841 | 2.8795±1.9476 | 0.344403 |
| **Cluster 3** |  |  |  |  |  |  |  |  |
| ParaHippocampal_R | 25.4597 | -29.343 | -19.3643 | 537 | 56.403 | 3.0878±3.1534 | 3.197±1.4699 | 0.016881* |
| Lingual_R | 19.2977 | -42.3252 | -9.3919 | 88 | 20.6807 | 2.9148±3.164 | 3.0123±1.116 | 0.025990* |
| Fusiform_R | 33.1836 | -24.4048 | -23.6994 | 164 | 34.5768 | 3.2794±3.089 | 3.3488±1.5129 | 0.124301 |
| **Cluster 4** |  |  |  |  |  |  |  |  |
| Parietal_Sup_L | -14.0252 | -75.6957 | 49.8369 | 213 | 19.7202 | 2.7299±4.3779 | 2.8001±1.3235 | 0.161642 |
| Calcarine_L | -5.40405 | -92.3792 | 13.8462 | 52 | 13.8257 | 2.6645±4.0751 | 2.6641±0.9656 | 0.992041 |
| Precuneus_L | -9.00341 | -73.7427 | 50.9558 | 318 | 24.8402 | 2.8978±4.1656 | 2.955±1.014 | 0.229131 |
| Cuneus_L | -15.8852 | -84.4441 | 40.4246 | 468 | 25.1561 | 2.5917±3.7369 | 2.6403±0.8701 | 0.278333 |
| Occipital_Sup_L | -18.8657 | -85.7333 | 39.5519 | 475 | 23.5537 | 2.5961±2.7484 | 2.6549±1.2669 | 0.161642 |
| Occipital_Mid_L | -29.1842 | -80.6128 | 19.4638 | 4 | 8.073 | 2.7555±1.7603 | 2.7582±1.4198 | 0.944333 |
| **Cluster 5** |  |  |  |  |  |  |  |  |
| Frontal_Sup_L | -25.2237 | 10.6073 | 52.9062 | 5 | 7.3627 | 3.0227±3.588 | 3.1444±2.199 | 0.014809* |
| Frontal_Mid_L | -38.1107 | -2.3484 | 52.6089 | 189 | 12.0757 | 2.93±3.4393 | 3.0587±1.5516 | 0.006876* |
| Precentral_L | -56.1688 | 3.9382 | 30.2653 | 197 | 11.3665 | 2.8251±4.6786 | 2.9096±1.4917 | 0.106247 |

X, Y and Z were in MNI coordinates. For each cluster, we report the brain regions of the highest peak value. Cortical connectivity is expressed in mm. * indicates a significance of p≤0.05 uncorrected.
