# Supplementary material for: Cortical Structural Connectivity Alterations and Potential Pathogenesis in Mid-Stage Sporadic Parkinson’s Disease
Source: Front Aging Neurosci. 2021 May 31;13:650371. doi: 10.3389/fnagi.2021.650371 (PMC8200851; doi:10.3389/fnagi.2021.650371)
Supplement: Supplementary file 9 [file Table_9.DOCX]

Supplementary Table 9 Brain regions of abnormal cortical connectivity in sPD patients versus control in seed 7

| Brain regions of abnormal cortical connectivity | Coordinates | | | Voxel | Peak F  score | Mean cortical  structural connectivity | | P-value |
| --- | --- | --- | --- | --- | --- | --- | --- | --- |
|  | X | Y | Z |  |  | sPD | NC |  |
| **Cluster 1** |  |  |  |  |  |  |  |  |
| Calcarine_L | -8.41183 | -92.5431 | -3.20726 | 1102 | 411.8536 | 2.5053±2.5791 | 2.6089±0.7464 | 0.007699* |
| Lingual_L | -9.67694 | -84.8585 | -11.3099 | 571 | 75.7894 | 2.7316±1.9824 | 2.8088±0.7633 | 0.027304* |
| Precuneus_L | -18.2168 | -47.8999 | -1.50968 | 22 | 9.8908 | 2.893±2.9363 | 2.9378±1.3588 | 0.300848 |
| Cuneus_L | -6.32676 | -73.1518 | 15.524 | 622 | 137.3279 | 2.6463±2.483 | 2.6889±0.642 | 0.248811 |
| Occipital_Sup_L | -15.82668 | -102.5578 | 1.965368 | 38 | 28.3538 | 2.3163±3.5477 | 2.3243±1.0916 | 0.857517 |
| Occipital_Inf_L | -22.7237 | -91.2275 | -14.658 | 31 | 18.3413 | 2.6155±3.0133 | 2.6785±1.3006 | 0.150188 |
| Occipital_Mid_L | -22.33791 | -100.7341 | -4.585351 | 44 | 17.6927 | 2.3408±3.8408 | 2.3908±1.2117 | 0.287195 |
| **Cluster 2** |  |  |  |  |  |  |  |  |
| Calcarine_R | 8.73196 | -83.4494 | 8.16601 | 960 | 46.1786 | 2.5811±3.2399 | 2.679±0.9954 | 0.024731* |
| Lingual_R | 20.3136 | -61.6651 | 4.10737 | 278 | 24.1787 | 2.7411±3.2341 | 2.8226±0.9279 | 0.057707 |
| Precuneus_R | 19.7339 | -62.5681 | 16.9486 | 16 | 13.4661 | 2.8793±2.4479 | 2.9764±1.5882 | 0.022373* |
| Cuneus_R | 15.9697 | -66.433 | 10.625 | 473 | 37.7013 | 2.5991±2.5602 | 2.6837±1.1607 | 0.038245* |
| Occipital_Sup_R | 21.3752 | -99.8809 | -8.57645 | 38 | 16.986 | 2.4028±4.2009 | 2.5313±2.1422 | 0.016023* |
| Occipital_Inf_R | 25.7757 | -98.3998 | -8.06218 | 69 | 15.0575 | 2.5437±3.4639 | 2.6454±1.8178 | 0.036481* |
| Occipital_Mid_R | 24.2994 | -99.2019 | -7.84047 | 4 | 14.6085 | 2.4182±3.8903 | 2.5518±2.0714 | 0.010163* |
| **Cluster 3** |  |  |  |  |  |  |  |  |
| Frontal_Sup_R | 18.3065 | 63.4742 | 14.1337 | 252 | 26.583 | 2.9953±2.0764 | 3.0377±1.7702 | 0.300848 |
| Frontal_Mid_R | 24.2882 | 56.1395 | 14.4411 | 4 | 7.7973 | 2.9564±2.5583 | 2.9721±1.5772 | 0.712165 |
| Frontal_Sup_Medial_R | 13.1097 | 63.4163 | 18.4829 | 270 | 21.0655 | 3.2838±2.2995 | 3.3391±1.6614 | 0.186544 |
| Cingulum_Mid_R | 5.83918 | 46.9944 | 32.3625 | 14 | 8.3406 | 3.5702±2.3668 | 3.6101±1.7233 | 0.344403 |
| Cingulum_Ant_R | 5.9114 | 47.4243 | 31.0968 | 3 | 7.8929 | 3.5223±2.3176 | 3.5624±1.6493 | 0.339371 |
| **Cluster 4** |  |  |  |  |  |  |  |  |
| Rectus_R | 4.85568 | 47.8764 | -17.5628 | 76 | 16.3309 | 3.1158±1.4692 | 3.1871±1.1002 | 0.035625* |
| Frontal_Mid_Orb_R | 6.04218 | 47.5741 | -12.9435 | 223 | 19.0525 | 3.2045±1.6188 | 3.2823±1.2242 | 0.029385* |
| Frontal_Sup_Medial_R | 10.16 | 49.1039 | -4.37977 | 63 | 11.8381 | 3.2424±2.5723 | 3.3323±1.6968 | 0.039155* |
| Cingulum_Ant_R | 10.4933 | 49.5378 | -1.6243 | 42 | 11.2999 | 3.3205±2.3828 | 3.3977±1.8747 | 0.068734 |

X, Y and Z were in MNI coordinates. ROIB is the regions of interest brain. For each cluster, we report the brain regions of the highest peak value. Cortical connectivity is expressed in mm. * indicates a significance of p≤0.05 uncorrected.
